# Gecko-Inspired Slant Hierarchical Microstructure-Based Ultrasensitive Iontronic Pressure Sensor for Intelligent Interaction

**DOI:** 10.34133/2022/9852138

**Published:** 2022-06-14

**Authors:** Yongsong Luo, Xiaoliang Chen, Hongmiao Tian, Xiangming Li, Yangtianyu Lu, Yang Liu, Jinyou Shao

**Affiliations:** ^1^Micro- and Nano-Technology Research Center, State Key Laboratory for Manufacturing Systems Engineering, Xi'an Jiaotong University, Xi'an 710049, China; ^2^Frontier Institute of Science and Technology (FIST), Xi'an Jiaotong University, Xi'an 710049, China

## Abstract

Highly sensitive flexible pressure sensors play an important role to ensure the safety and friendliness during the human-robot interaction process. Microengineering the active layer has been shown to improve performance of pressure sensors. However, the current structural strategy almost relying on axial compression deformation suffers structural stiffening, and together with the limited area growth efficiency of conformal interface, essentially limiting the maximum sensitivity. Here, inspired by the interface contact behavior of gecko's feet, we design a slant hierarchical microstructure to act as an electrode contacting with an ionic gel layer, fundamentally eliminating the pressure resistance and maximizing functional interface expansion to achieving ultrasensitive sensitivity. Such a structuring strategy dramatically improves the relative capacitance change both in the low- and high-pressure region, thereby boosting the sensitivity up to 36000 kPa^−1^ and effective measurement range up to 300 kPa. To verify the advantages of high sensitivity, the sensor is integrated with a soft magnetic robot to demonstrate a biomimetic Venus flytrap. The ability to perceive weak stimuli allows the sensor to be used as a sensory and feedback window, realizing the capture of small live insects and the transportation of fragile objects.

## 1. Introduction

Flexible pressure sensors have attracted much attention due to the capability to sense external stimuli and provide sensory feedback for the applications of intelligent soft robot, human-machine interfaces, and wearable electronics [1–10]. Endowing these systems with perceptive ability can significantly enhance their autonomous task capabilities. For instance, sensors were embedded in a pneumatic octopus hand, which enabled it to detect its own posture and obtain information about the target object during the grasping process [4, 11, 12]. The sensor integrated on the wearable glove can be used to reconstruct the hand movement and provide the tactile information and then be used in the field of rehabilitation medicine, virtual, and augmented reality [13–16]. However, limited by not very high sensitivity and resolution, most of these reported sensors are unable to realize the perception of fine motion and slight touch, which cannot satisfy the requirement of friendliness and safety for intelligent systems in the interacting process [17, 18]. In order to maintain safety during the interaction, it is important for perceptions to detect weak and tiny external stimuli and provide feedback information rapidly, synchronously, and continuously during, and thus, the sensors used for perception are required to possess outstanding sensing properties, such as sensitivity and limit of detection (LOD) [19].

Engineering the functional layer with types of microstructures such as microcones, microdomes, micropillars, and rough surface to decrease the compression stiffness is recognized as an effective way to improve the properties of the flexible pressure sensors [20–27]. Mannsfeld's group introduced a micropyramid structure to construct capacitive sensors and obtained good sensitivity by using the easy compressibility of the pyramid tip [28, 29]. Li and coauthors reported a hierarchical nanonetwork structured piezoresistive pressure sensor composed of spun fibers and conductive low-dimensional materials, achieving a high sensitivity and wide detection range [30]. Wang and coauthors demonstrated a ferroelectret generator with functional materials-coated fabric, which exhibited strong piezoelectricity [31]. In addition to traditional capacitive, piezoelectric, and piezoresistive types, in recent years, the development of ionic gel has provided a new way for constructing pressure sensors [32–38]. Iontronic pressure sensors are constructed by electric double layers (EDLs) formed at the contact interface of ionic gel and conductive medium. The electron double layers at the dielectric/electrode interface exhibit remarkable evaluation due to the atomic scale distance (~1 nm) between the positive and negative charges, fundamentally liberating the upper limit of capacitance value and providing the possibility to significantly promote the sensitivity and resolution of capacitive sensors [34, 35, 39]. By changing the working principle from the thickness reduction to contact area expansion, iontronic pressure sensors have already demonstrated substantially improved sensitivity. The expansion form of the EDL interface has been optimized by structuring the ionic layer through the above topology design including microcones, microdomes, and textile surface, in which the top of the microstructure is gradually pressed flat, resulting in the contact area growth [33, 40–48]. Specifically, in the structural strategy that relies on axial compression deformation, when the intimate contact between the microstructure and the counter layer forms, the deformation of the functional layer will become more and more difficult due to the increased pressure resistance of the material itself during the compression process, which makes it difficult to further flatten the structure to contact the electrode, thus leading to a poor sensitivity. Recently, there have been some attempts to break through the growth stiffness brought by axial compression by introducing upright pillars with high aspect ratios [36, 49]. When the pressure increases to a certain level, according to Euler's formula, the buckling instability occurs. This process softens the stiffness and brings a great increase in sensitivity. It should be pointed out that this process just occurs after the axial compression, and it cannot avoid the poor sensitivity in the beginning. Meanwhile, buckling instability is triggered by the small perturbation, whose direction is actually random, thereby inevitably introducing contingency caused by buckling instability, which will affects the repeatability and reliability of the sensor. Moreover, in the process of flattening the structure, the ionic layer gradually conforms to the electrode surface. The existing research basically only focuses on making the structure more deformable but does not consider the efficiency of transforming deformation into functional interface growth. Limitation of the contact area restricts the further improvement of the sensing properties. How to furthest enlarge the increasing trend of the EDLs interface area under pressure is crucial for construct highly sensitive iontronic pressure sensor.

Inspired by gecko's feet, which consist of hierarchical slant scales and setae to increase the adhesive area and thus the van der Waals force to crawl on steep walls, here, we introduce a slant hierarchical microstructure to act as an electrode contacting with an ionic gel layer to construct a new iontronic capacitive pressure sensor with maximum interface expansion. The slant architecture undergoes designed bending deformation, which has stronger deformability than compression and exactly avoids the influence of the pressure resistance on stiffness during the deformation process. Meanwhile, the microbeads on the slant scales can be embedded into the hyperelastic gel under pressure to enhance the contact interface area growth, synergistically contributing to the increase in the capacitance brought by EDLs. The sensor enhanced by this hierarchical structure of slant scales and beads on the scales is able to achieve an ultrahigh sensitivity of 36000 kPa^−1^ and an ultralow limit of detection of 0.015 Pa, which far outclass the reported capacitive pressure sensor to the best of our knowledge. In addition, the secondary beads also provides a lager deformation space for area growth compared to the slant flat scales, thus leading to a lager effective measurement range at least up to 300 kPa. Such amazing sensing performance is able to completely detect a wide range of pressures from sound waves to heavy objects and perfectly reproduce the damped oscillation of a water droplet. And this sensor was demonstrated to act as an ultrasensitive sensory and feedback window for biomimetic Venus flytrap to guarantee the safety and friendliness during the interaction, realizing the capture of small live insects and the transportation of fragile objects.

## 2. Results

### 2.1. Design Principle of Iontronic Capacitive Sensor with Bioinspired Slant Hierarchical Microstructure

**Figure 1:**
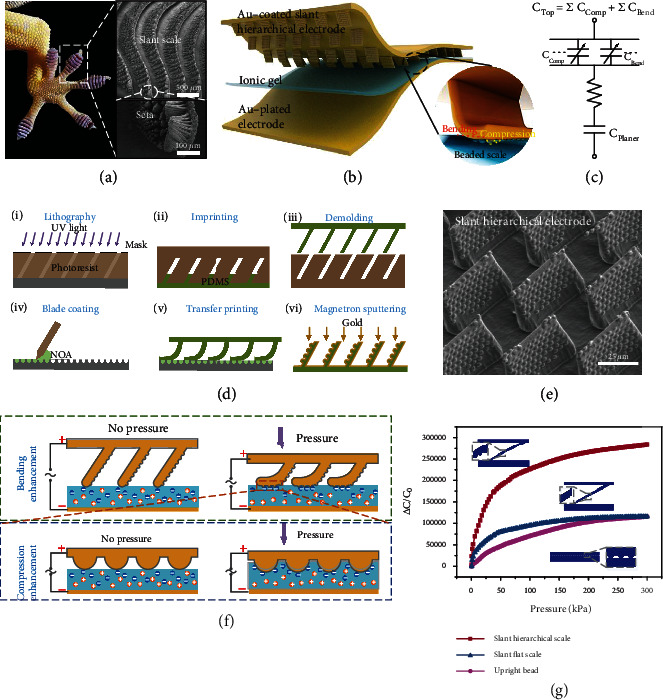
Design of the iontronic capacitive pressure sensor. (a) Gecko's feet consisting of slant scales (scale bar, 500 *μ*m) and setae (scale bar, 100 *μ*m) to increase the adhesive surface. (b) Schematic illustration of the iontronic sensor with slant hierarchically structured electrode and ionic gel layer. (c) Equivalent electrical circuit of the sensor. (d) Manufacturing process of the slant hierarchical electrode. (e) SEM images of the structured electrode, the scale bar is 25 *μ*m. (f) Bending enhancement of slant scales and compression enhancement of beads on contact area change at the interface. (g) Comparison of capacitance response of three different microstructures, including slant hierarchical scale, upright bead, and slant flat scale.

In nature, the gecko can crawl on steep walls, which depends on its feet consisting of parallel-arranged slant scales and the setae on them (Figure 1(a)). The slant scales of the feet reduce the stiffness when it attaches to the wall, and the hierarchical setae maximize the contact area to enhance the van der Waals force [50]. As mentioned above, improving the relative change of the area is the key to boost the sensitivity of iontronic capacitive sensor. The slant hierarchical structure that is easy to flatten and has a large surface area is an ideal type for constructing highly sensitive iontronic pressure sensors. Inspired by gecko's feet, we introduce a slant hierarchical microstructure to act as an electrode contacting with an ionic gel layer to construct a new iontronic capacitive pressure sensor (Figure 1(b)). The capacitance change is mainly induced by the change in the contact area at the interface between the slant hierarchical electrode and the ionic gel layer. As the inset shows, the structured electrode appears as a hierarchical structure, which consists of slant scales and beads on the scales. When subjected to an external force, the slant scales bend, resulting in a longer contact line in the length direction; and because of the hyperelasticity of ionic gel, the microbeaded structures on the slant scales are embedded in the gel, which causes the contact line in the width direction to become longer as well, therefore increasing the contact area from two dimensions. The capacitance of the entire sensor is equivalent to the EDL capacitors generated at the electrode/electrolyte interface with a bulk resistance connected in series, as shown in the equivalent circuit model of Figure 1(c). The EDL capacitors are divided into two parts, namely, the fix capacitor at the interface between the plane electrode and gel layer and the variable capacitance at the interface between the structured electrode and gel layer. The variable capacitance comes from the bending of slant scales and the compression of microbeads on the scales. For capacitors in series, the total capacitance *C* is given by 1/*C* = 1/*C*_planer_ + 1/*C*_top_, where *C*_planer_ and *C*_top_ denote the capacities brought by the planer electrode and the microstructured electrode, respectively. Under pressures, the contact interface between the planer electrode and the ionic gel does not change, so *C*_planer_ is a fixed value. Therefore, the total capacitance *C* is positively correlated with the *C*_top_, and thus, the sensitivity of the proposed sensor is observably contributed by the area change caused by the bending of the slant scales and the compression of the microbeads on the scales.

The manufacturing process of the bioinspired slant hierarchical electrode was shown in Figure 1(d). Slant structure template was obtained by oblique lithography, and then, the primary structure of slant scales was prepared by imprinting. Adjusting the incident angle of ultraviolet light during photolithography can obtain a template with customized tilting angle. The slant scale was pressed on the secondary structure template filled with the blade-coated material to complete the transfer imprinting, after which gold was deposited on the surface as a structured electrode. It is worth noting that the prepressure in the transfer printing process is very important. Too high or too low pressure will cause structural defects, and the transfer printing effect under different prepressures can be found in Figure S1. The obtained structure was a perfect replica of the template as the scanning electron microscopy (SEM) image in Figure 1(e) shows, displaying uniformity different from that of random structure and thus providing structure guarantee for maintaining stability. The manufacturing method of imprinting and transfer printing can be also used to prepare a large-aspect-ratio hierarchical structure closer to the gecko's feet and is widely applicable to the controllable manufacturing of various hierarchical structures (Figure S2). The ionic gel was prepared using a blended solution of polyvinyl alcohol (PVA) as the polymer matrix and [EMIM][OTF] as the ionic liquid. [EMIM][OTF] has been selected because it is a nontoxic water-soluble ionic liquid that does not react with water to produce hydrofluoric acid [38]. The capacitance value per unit area brought by the material configuration is detailed in Figure S3.

Initially, when the sensor is uncompressed, there is only top contact between the slant scales and the gel. As the slant structure bends under the application of pressure, the tops of the scales slide and the sides gradually come into contact with the gel, causing the contact line in length direction to grow, as shown in the side view of the scales (Figure 1(f)). Instead of being limited by Poisson's ratio of the material itself under direct vertical compression, the bending behavior has a very significant advantage of eliminating the pressure resistance and makes it easy to generate a lager contact area change [13]. In the process of contact between the sides of the slant scales and the ionic gel, the microbeads on the scale are gradually embedded into the hyperelastic gel under the pressure. The contact state changes from the initial discrete point contact to the hemispherical surface contact, and finally the gel conforms to the beads, not only reducing the initial contact area but also increasing the maximum contact area during the deformation process. Consequently, as shown in Figure 1(g), such a structural method combining slant scale and microbead, whose sizes are, respectively, almost the same as that in the slant flat scale and that in the upright bead, has the potential to substantially increase the relative change in contact area, as a result, the relative capacitance change. Moreover, due to the pressure resistance of the microbeads and the gel, the saturation pressure for the contact area change is greater than that of the slant flat scale, thus leading to a larger effective measurement range.

### 2.2. Mechanism of Contact Area Growth

**Figure 2:**
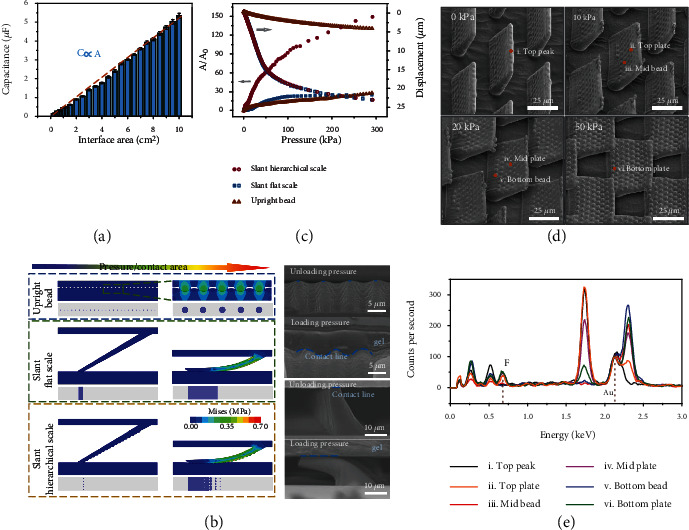
Mechanism of contact area growth. (a) The proportional relationship between capacitance brought by EDLs and the contact interface area. (b) Contact process of different structures with increasing pressure, including upright beads, slant scales, and slant hierarchical scales, the top two scale bars are 5 *μ*m and the bottom two scale bars are 10 *μ*m. (c) Numerical simulation results of the contact area change and the displacement of the upper electrode as functions of pressure. (d) SEM images showing the deformation process of the slant hierarchical microstructures under different pressures, the scale bars are 25 *μ*m. (e) Elemental analysis of different domains on the hierarchical scales to determine the contact state with ionic gel.

The capacitance value of the EDLs is closely related to the contact area at the gel/electrode interface, and the capacitance value is directly proportional to the area through experimental methods, as shown in Figure 2(a). Therefore, the mechanism of capacitance change and growth is consistent with that of contact area. The essence of studying capacitance change is to study the contact area change.

To understand the mechanism of contact area growth, finite-element analysis (FEA) was applied to investigate the deformation of three representative microstructures, including upright beads, slant flat scales, and slant hierarchical scales. The size of the sheet in the slant hierarchical scale is similar to that in the slant flat scale, and the size of the beads in the slant hierarchical scale is similar to that in the upright bead. The initial contact state and the process contact state and the contact projection were shown in Figure 2(b). In the initial state, compared to the uniform contact on the entire surface of the upright beads and the continuous line contact of the slant flat scales, the hierarchical structure with protruding spheres at the front possesses discrete point contact and has the smallest initial contact area. During the deformation process, the hierarchical architecture can be bent easily as the slant flat scale to increase the contact area, which can be further enhanced by the beads on the surface. Benefiting from the above two points, the combined architecture has a greater relative area change (*A*/*A*_0_) during the deformation process. The deformation processes of the slant scale and bead were consistent with the simulation, as shown in the SEM images. The numerical results obtained by the simulation were shown in Figure 2(c). Since the slant hierarchical scales have the smallest initial contact area and the largest variable contact area, it has the largest relative contact area change. Due to the liberation of the pressure resistance brought by the slant structure, it is easier to drive the displacement of the upper electrode, and thus, the relative contact area changes of the slant structures are generally greater than that of the upright structure. It should be further pointed out that due to the enhanced roughness of the electrode surface after oxygen plasma treatment, the actual contact area between the ionic gel and the electrode will be much larger than the simulation result (Figure S4). Moreover, the initial contact area value in the simulation was obtained under a small step size (~100 Pa). Although this pressure was very small, it has actually caused a part of the interface contact, which also caused the relative area change in simulation smaller than that in experiment. Even if there are some differences in absolute values, the trend of sensor performance in the simulation and experiment is consistent, and the performance comparison of the three sensors is very obvious, which can be used to illustrate the significant effect of designing a slant hierarchical structure on performance improvement.

In order to verify that the deformation process of the slant hierarchical scales was consistent with the simulation, the deformation shape of the structure under different pressure levels was observed, as shown in Figure 2(d). The observation method was to use the UV glue to memorize the deformation of each pressure stage and then obtain the samples through molding the UV template (Figure S5). The highest part of the slant hierarchical structure in the observation result was the part in contact with the plane. Consistent with the simulation results, the top of the slant scales slipped and bended after being pressed, and the contact line increased. And the slant flat scales displayer the same process (Figure S6). From the SEM images, it was impossible to judge whether the ionic gel was ideally embedded among the beads. However, it is worth noting that after the slant hierarchical scales contact the ionic liquid, a part of the ionic liquid would remain more or less on the surface. Therefore, we performed elemental characterization of different domains after pressing the slant electrode on the ionic gel with corresponding pressures. The characterization domains were the top, middle, and bottom parts of plate and bead. The fluorine element unique to the ionic liquid but not available in the electrode was used as a marker, and the gold element on the electrode surface was used to normalize the counts per second to obtain the element analysis of each domain under different pressures (Figure 2(e)). It can be known from the distribution of fluorine that points of i, iii and v had not been in contact with the ionic gel, while points of ii, iv and vi had residual fluorine. It can be judged that as the pressure increased, the contact interface expanded from the scale top to the bottom, and from the bead top to the bottom.

### 2.3. Capacitive Response Properties of the Iontronic Sensor

**Figure 3:**
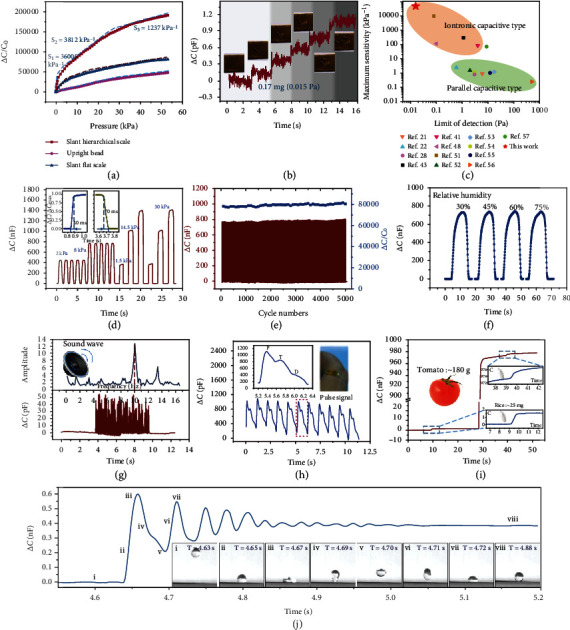
Typical characteristics of the pressure sensor. (a) Capacitance response in pressure region from 0.1 Pa to 53 kPa. (b) Stepped increase associated with the accumulation process of five dandelion seeds on the sensor. (c) Comparison of the maximum sensitivity and LOD between the proposed sensor and capacitive pressure sensors reported in the literature. (d) Repeatable response under periodical pressures of 1.5 kPa, 2 kPa, 8 kPa, 14.5 kPa, and 30 kPa. (e) Durability test results after 5000 loading and unloading cycles at a pressure of 10 kPa, showing no degradation in the relative capacitance change. (f) The stable capacitance response under pressure of 10 kPa in the environment with the humidity of 30%, 45%, 60%, and 75%. (g) Capacitance response of the sensor to sound waves of 80 dB generated form a conventional speaker. (h) Human pulse signal curves tested by the sensor attached to the wrist. (i) Detection of micropressure under low and high pressures. (j) Capacitance response to a fallen water droplet of 10 *μ*L, with the insets depicting the water droplet falling process.

The electrical characteristics of the fabricated iontronic pressure sensor based on slant hierarchical electrode were tested to verify the effectiveness and feasibility of the proposed strategy and analysis. Benefiting from the bending deformation, the slant scales displayed sharp increase in capacitance. Moreover, since the beads reduced the initial contact area and increased the process contact area, the combined structure had a greater capacitance change. The pressure sensitivity *S* is defined as *S* = *δ*(Δ*C*/*C*_0_)/*δP*, where *P* denotes the applied pressure and *C* and *C*_0_ denote the capacitance with and without pressure, respectively. A high sensitivity means that small changes in the pressure induce a large capacitance change. In general, iontronic capacitive pressure sensors exhibit ultralarge capacitance changes, which can reach up to 10^6^ times the value of *C*_0_, far greater than what noniontronic sensors exhibit. The proposed sensor with slant hierarchical scales demonstrated an ultrahigh pressure sensitivity of 36000 kPa^−1^ in the pressure range of 0.1 Pa-1.2 kPa, 3812 kPa^−1^ in the pressure range of 1.2-25 kPa, and 1237 kPa^−1^ when the pressure was above 25 kPa (Figure 3(a)). The sensor with slant flat scales displayed a pressure sensitivity of 8148 kPa^−1^ (0-3.5 kPa), 2125 kPa^−1^ (3.5-15 kPa), and 975.3 kPa^−1^ (>15 kPa), and the maximum pressure sensitivity of the sensor with upright beads was 1212 kPa^−1^. Remarkably, the sensitivity of the proposed sensor is the highest among all reported EDL capacitive pressure sensors. It IS worth emphasizing that the structuring strategy and contact interface expansion mechanism proposed here are not only valid for EDLs-iontronic capacitive sensors and should be useful for other capacitive system. Therefore, in order to increase the importance and universality of the proposed method in improving the sensor performance, a new pressure sensor based on pseudocapacitance using the same strategy was constructed (Figure S7). Since the microstructure is directional, the capacitance responses under different tangential forces should be different, and the sensing performances have also been characterized, as shown in Figure S8. In the bending state, the structural layer of the sensor will be squeezed and deformed, resulting in an increase in the capacitance value, thereby reducing the relative capacitance change. Therefore, for different installation and fixing methods, the sensor needs to be calibrated again before use (Figure S9).

Benefiting from the slant hierarchical microstructure, this sensor has such an ultrahigh output-input ratio; therefore, it has great potential for smaller detection work. Furthermore, the LOD of the sensor was investigated by placing a sequence of dandelion seeds on it (Figure 3(b)). The capacitance response revealed a stepped increase associated with the accumulation process of five dandelion seeds on the sensor. The insets in Figure 3(c) demonstrate the process of gradually placing five seeds on the sensor and correspond to each step on the curve. The average mass of the dandelion seeds was 0.17 mg, which corresponds to a very low pressure of only 0.015 Pa. Such a small LOD and resolution have never been reported in the existing literature. As summarized in Figure 3(c), the proposed slant hierarchical iontronic capacitive pressure sensor exhibited an unprecedented ultrahigh maximum sensitivity (36000 kPa^−1^) and ultralow LOD (0.015 Pa), which, to the best of our knowledge, surpasses all the existing iontronic capacitive and parallel capacitive sensors reported in the literature [21, 22, 28, 41, 43, 48, 51–57]. In different pressure ranges, the proposed sensor also showed the highest sensitivity. Such high performance is not only a major breakthrough in the field of capacitive pressure sensors but also the entire field of pressure sensors (Figure S10). The sensitivities of the iontronic capacitive pressure sensors in the green box are significantly (orders of magnitude) higher than those of the noniontronic sensors in the yellow box, and the same applies for the LOD. The sensitivity of the proposed sensor is 10 times higher than the highest sensitivity of the most recently reported iontronic sensors, and the LOD is only 1/5 of the reported lowest value. It is worth noting that since the electrode was manufactured using the molding process, the size and arrangement of microstructures can be adjusted to customize the sensing performance. The sensitivity can be further enhanced by adjusting the slant angle of the hierarchical structure, the size of the slant scale, and the effective number of scales and beads per unit area. For example, when the structure is the same but the inclined angle is different, the closer the initial state of the slant scale is to the horizontal, the easier it is to be flattened; and when the angle is constant, the thinner and higher the scales are, the easier they are to be bent. This more deformable structure will bring a greater growth in contact area and thus a higher sensitivity. When the width of the slant scales or the numbers of scales or beads per unit area increase, the electrode surface that contributes to the contact interface will be more, and the sensitivity will be higher.

In addition, benefiting from the controllable manufacturing, the sensor with uniform structure displayed good stability and durability for long-term and/or cyclic. After a large number of loading cycles, the structured electrode was still stable, and the conductivity degraded slightly. Periodical pressures of 2 kPa, 8 kPa, 1.5 kPa, 14.5 kPa, and 30 kPa were applied on the sensor, and the response curves presented remarkable repeatability, ensuring a consistent and stable performance of the sensor during actual use (Figure 3(d)). A constant of 86.5% was used as the standard time value of the pressure sensors and a pressure of 5 kPa was applied to the sensor. The sensor exhibited a response time of ~40 ms and a reset time of ~70 ms as shown in the inset, which are close to the perception speed of the human skin (30-50 ms). Actually, the response and reset times depend on the testing platform, and the excitation closer to the ideal step signal can get a faster dynamic response. When a periodic pressure is applied by an exciter with a faster loading rate, the sensor can achieve a saturation response at the frequency exceeding 10 Hz. The sensor can respond to the excitation at the frequency of 200 Hz, but the output amplitude will be attenuated compared with the frequency response within 10 Hz (Figure S11). Moreover, the manufacturing process is photolithography, ICP etching, and imprinting. The advantage of this method is that it can ensure the accuracy and consistency of the microstructure in different batches, and thus, the sensors in different batches have highly consistent structures with the same height. Therefore, polyimide (PI) tape with the same thickness can be used as the spacers to ensure that the sensors have the same initial capacitance (*C*_0_) and repeatable sensing performance (Figure S12). The capacitive sensor endured 5000 loading-unloading cycles at a pressure of 10 kPa and frequency of 0.5 Hz. Although cracks occurred on the surface of the electrode, due to the constraints of the discrete slant scales and the enhanced adhesion process, the conductive coating on the electrode surface will not fall off, thereby maintaining good conductivity (Figure S13). The stability of the electrode further ensured the stability of the capacitance response, and the response curve during 5000 cycles is plotted as Figure 3(e). The entire output signal exhibited little degradation after 5000 cycles, and in each cycle, the response was complete and consistent, showing little variation in the relative capacitance change. Because the sensor was sealed by tape, the ionic gel was isolated from the environment and thus humidity had little influence on capacitance (Figure 3(f)). For unsealed sensor, the ionic gel will absorb moisture from the high-humidity environment, resulting in the changes in electrical properties and thus capacitance. Excessive water will dissolve the ionic gel layer, causing the contact between the upper and lower electrodes and then a short circuit (Figure S14). This sealing method can not only ensure that the ionic gel does not absorb moisture from a high humidity environment but also ensure that it will not lose moisture and dry up in normal temperature and humidity, so that the sealed sensor has better stability and consistency in long-term use (Figure S15). Under different pressures, the effects of temperature on capacitance response are presented in Figure S16.

### 2.4. Broad Range Pressure Sensing

The slant hierarchical structure endows the sensor with ultrahigh sensitivity in a wide pressure range. Ultrahighly sensitive capacitive pressure sensor makes it possible and convenient to efficiently detect a wide range of pressures from millipascals to kilopascals. The sound waves (~80 dB) generated by the speaker traveled through the air at a distance of 2 cm and then arising vibration on the sensor, which can be successfully converted into the capacitance signals, as shown in Figure 3(g). The signal amplitude can qualitatively reflect the strength of the sound wave, and within the range of response time and recover time of the sensor, the frequency of the sound wave can be effectively distinguished. Human pulse signal is composed of three component waves that include percussion wave (PW), tide wave (TW), and dicrotic wave (DW) [19, 20]. The proposed sensor can perfectly reproduce the pulse signal and clearly identify these characteristic peaks, as shown in Figure 3(h). In addition, these clearly visualized signal information can be used to diagnose the patient's cardiovascular and cerebrovascular health, etc. In addition to showing ultrahigh-pressure resolution in a small pressure range, the ideal sensor should still maintain a high resolution over a wide pressure region in actual use. As mentioned above, the proposed sensor can clearly detect a very tiny change in pressure (0.015 Pa) under low pressures; and under large pressures, it can still effectively identify the tiny pressure change (Figure 3(i)). For the test, a grain of rice (~25 mg, which corresponded to an effective pressure increment of *Δ*P~5 Pa), a tomato (~180 g, which corresponded to an effective pressure increment of *Δ*P~36 kPa), and a grain of rice was placed on the sensor in sequence, and the capacitance response was recorded throughout the process. The capacitance response in the low pressure region was as expected, and the pressure increment after loading the pressure of 36 kPa successfully led to a stepped escalation of the capacitance. It should be noted that in different pressure regions, the micropressures that the sensor can identify are not the same due to the different sensitivities in different pressure regions. In the low-pressure range (<1 kPa), the sensitivity of the sensor is ultrahigh, so it has a clearer response to extremely small micropressures. However, in the high-pressure range, the sensitivity of the sensor decreases, and the minimum micropressure that can be detected is far from reaching the LOD. In the pressure range exceeding 20 kPa, the minimum pressure that can be recognized is about 2.2 Pa (Figure S17).

Due to its extremely high sensitivity and good dynamic response, this sensor is very suitable for detecting weak and continuously changing external signals, such as collisions and vibrations of water falling on the sensor surface. To evaluate the rapid response of the sensor to dynamic force, a water droplet of 10 *μ*L was dropped on its superhydrophobic surface from a height of one centimeter. The superhydrophobic surface was insitu prepared on the outer surface of the sensor by uniformly coating the precured polydimethylsiloxane (PDMS) layer with micronanoparticles. The detailed preparation process of the superhydrophobic surface can be found in the supporting information (Figure S18). Due to surface tension and elasticity, when the water landed on the superhydrophobic surface, it bounced and oscillated as shown in Movie S1. This process was very rapid, and due to the small mass of the water droplet, the force exerted during this process was extremely weak. As it can be observed in Figure 3(j), the capacitive output response was able to perfectly and completely capture the dripping, oscillating, and static states of the water droplet, as shown in insets. The good reproduction of the oscillation process proves that the sensor can be effectively applied to detect small moving objects, which is exactly in line with the function of the Venus flytrap to sense live insects.

### 2.5. Biomimetic Venus Flytrap with Iontronic Sensor as Trigger

**Figure 4:**
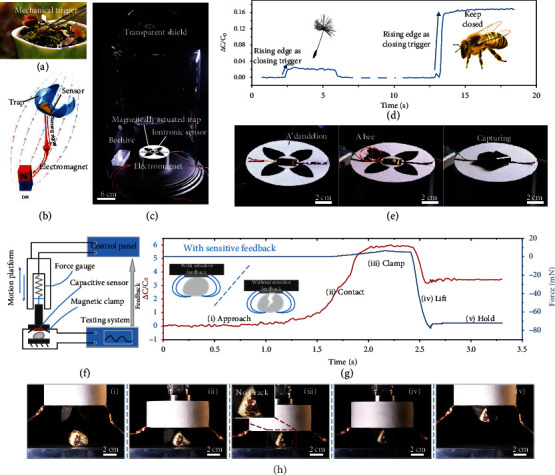
Intelligent autonomous system using the sensor as the sensory and feedback window. (a) Images of the Venus flytrap and the leaf closure triggered by mechanical stimuli. (b) Schematic diagram of the biomimetic Venus flytrap sensing the stimuli and closing the trap. (c) Self-built experimental flying object automatic capture device. (d) Capacitance response used as the feedback signal for automatically capturing objects. (e) Biomimetic Venus flytrap passively capturing a dandelion and a bee, respectively, that fall on its surface. (f) Schematic diagram of the active intelligent grabbing system. (g) Capacitance and force change during the process of the gripper successfully grabbing a quail egg without any damage, inset showing the nondestructive grabbing of quail egg under ultra-sensitive feedback compared with that without sensitive feedback. (h) Showcase of grabbing process.

The proposed sensor is able to quickly and accurately identify weak, dynamic, and continuous signals and can be integrated into the sensory system of the intelligent robot to interact with the outside world in real time. Due to the extremely high sensitivity and extremely low LOD of the sensor, the pressure limit that can be detected by the proposed sensor-based sensing system is one to two orders of magnitude lower than the existing research. And such a sensitive perception function can give the intelligent system a smaller neural threshold, so that the intelligent system can respond to smaller stimuli, providing a deeper guarantee for the safety and friendliness of human-machine interaction. Actually, we proposed a biomimetic Venus flytrap, in which the sensor acted as the mechanical trigger. In nature, the Venus flytrap has the fastest behavior in the plant kingdom (100-200 ms) [58–60]. More specifically, the closing of the insect trap is induced by a biochemical reaction transmitted by the setae after they sense the external mechanical stimuli (Figure 4(a)). In our autonomous biomimetic system, the iontronic sensor that plays the role of the trigger provides the biomimetic Venus flytrap with the ability to identify light touches or tiny objects. The capacitance signal induced by the external stimuli mimics the biological signal generated by the Venus flytrap trigger and controls the electromagnet to drives the flexible magnetic actuated trap to bend and complete the grasping (Figure 4(b)). The magnetic actuated trap was prepared by printing, the whole manufacturing process can be found in the supporting information (Figure S19). The magnetic actuated trap had faster response time than that of the Venus flytrap in nature and was able to grab larger objects, and the driving force of the magnetic trap can be adjusted by changing the voltage of the electromagnet (Figure S20). A transparent display device was built to observe the ability of the biomimetic Venus flytrap to capture flying objects (Figure 4(c)). The electromagnet under the flytrap was powered by a power supply circuit controlled by a relay, and the on-off function of the relay was controlled by the capacitance signal acquired through the signal circuit. When the electromagnet was turned on, the generated magnetic field led the magnetic gripper to close. When even a small object, such as a dandelion, fell on the sensor, the output signal (Figure 4(d)) exhibited an abrupt upward step, and then, the processor recognized the steeply ascending curve and turned on the power supply circuit to power the electromagnet, thereby applying a magnetic field that closed the magnetic film trap (Figure 4(e) and Movie S2). The time from the target contact the sensor to the completion of the capture was about 140 ms, which is equal to the sum of the response time of the sensor itself and the sampling, processing, and transmission time of the acquisition circuit and has nothing to do with the mass of the object. When the dandelion was removed from the trap, the output signal exhibited a downward step, and then, the processor recognized the descending curve and turned off the power supply circuit, thereby canceling the magnetic field and releasing the magnetic film trap. For moving small and agile insects such as bees, the iontronic sensor can still quickly and timely transmit feedback to complete the capture (Movie S3).

### 2.6. Protection for Fragile Object Handling

The autonomous biomimetic Venus flytrap with ultrasensitive trigger can be used as an intelligent gripper to grab delicate objects. When actively grasping an object, the sensor can sense the touch when the first contact occurs, and the triggered capacitance signal is utilized as a feedback to control the end of the motion platform to stop approaching behavior and close the magnetic trap. The schematic diagram of the device when grabbing objects was shown in Figure 4(f). The sensitive gripper and the electromagnet applying the driving magnetic field were fixed upside down on the end of the mechanical ram acting as the *z*-direction motion platform. When the robotic arm moved downwards, the trigger of the gripper was touched by the target to cause the capacitance signal change. Intelligent gripper with ultrasensitive feedback has such an advantage that when grabbing fragile objects such as strawberries and eggs, the sensor can promptly detect the arrival of the target and quickly complete the grab task, preventing the target object from being crushed caused by the gripper continuing to descend due to the lack of sensing feedback or the slowness of perception (Movie S4). The process of the gripper successfully grabbing a quail egg without any damage can be perfectly reflected by the capacitance and force curve (Figures 4(g) and 4(h)). Photos from i to v in the figure were the working stages of the intelligent gripper from approaching, contacting, clamping, and lifting to holding stably. When the sensor and the quail egg began to contact, the capacitive signal appeared a rising edge, which not only triggered the closing of the electromagnet circuit to generate a magnetic field to drive the trap but also acted as a cut-off signal to stop the robotic arm downwards and change to upwards. It can be seen that the rising edge of the force curve collected by the commercial force sensor with a resolution of up to 0.5 mN fixed on the robotic arm started later than that of the capacitance curve, indicating that an ultraweak force had actually been generated after the contact, which caused the iontronic sensor output changes, but the commercial sensor was somewhat dull to this change. When the feedback of the grasping system was relatively dull, the force can only be sensed until it was increased to a certain value. In this case, the pressure may have exceeded the pressure limit of the vulnerable object and result in damage (Figure S21 and Movie S5).

## 3. Discussions

In summary, a new bioinspired iontronic capacitive pressure sensor based on slant hierarchically microstructured electrode is demonstrated. The structuring strategy provides a combinatorial expansion for the gel/electrode contact interface from both the bending and compression deformation. Instead of being limited by Poisson's ratio of the material itself under direct vertical compression, the overall bending behavior of the slant scales has a very significant advantage of eliminating the pressure resistance and makes it easy to generate a lager contact area change. In the process of bending, the sides of the slant scales turn to contact the ionic gel, and the microbeads on the scale are gradually embedded into the hyperelastic gel under the pressure, with the contact state changing from the initial sharp point contact to the hemispherical surface contact. Such strategy not only reduces the initial contact area but also increases the maximum contact area during the deformation process, substantially increasing the relative change in contact area and, as a result, the relative capacitance change. Moreover, the beads on the scales allow the contact area growth potential in the large pressure region and thus a wide measurement range can be obtained, while the measurement range of slant flat scales is small since the contact area is close to saturation after bending.

Benefiting from this structural strategy, the proposed sensor was able to achieve an ultrahigh sensitivity of 36000 kPa^−1^, an ultralow limit of detection of 0.015 Pa, and a broad measurement range at least up to 300 kPa, which were significantly more outstanding than that of any reported capacitive pressure sensor. The dynamic speed of the sensor (~40 ms in respond and ~70 ms in reset) is on the same order of magnitude as that of human skin. Under loading and unloading cycles, the sensor produced repeatable and reproducible signals, whereas the capacitance response showed little degradation over 2000 cycles at a peak pressure of 10 kPa at 0.5 Hz. Such amazing sensing performance is able to perfectly and completely reproduce a wide range of pressures and the damped oscillation of a water droplet. This sensor has the further potential to act as a sensory and feedback window for intelligent systems such as a biomimetic Venus flytrap to provide the feedback for trap closing, which can be used to capture moving objects and protect fragile targets in handing.

## 4. Materials and Methods

### 4.1. Finite Element Analysis

FEA was performed using the commercial soft Abaqus 6.14. The structured top electrode was treated as an incompressible Neo-Hookean material with a height of 30 *μ*m and Young's modulus of 2 MPa. The width of a single slant scale was set to 5 *μ*m, and the angle with the horizontal plane was 45 degrees. The ion gel on the flat electrode was regarded as a hyperelastic structure with Young's modulus of 20 kPa and Poisson's ratio of 0.4. At the sliding interface between the slant scale structure and the bottom electrode, a friction coefficient of 0.1 was set. There was no penetration at all contact interfaces of the entire model. The apex of the bead at the front end of the slant scale was matched with the lower plane. Since the top bead and the electrode are in ideal point contact when there is no pressure, the contact area at this time is 0, which makes the relative area change difficult to determine. Therefore, at the beginning of the calculation, we gave a very small step size (~100 Pa), and the contact area obtained under this step size was set as the initial area.

### 4.2. Fabrication of PDMS Template

A positive photoresist mold was prepared by coating the photoresist AZ 4620 (10 mL; AZ Electronic Materials USA Corp) on a glass at 1500 rpm for 30 s, and exposing it to UV light under the mask after being heated at 90°C for 600 s. The slant lithography structure can be obtained by adjusting the angle of incident light, which was used to prepare the template required for the titled hierarchical electrode. PDMS (Sylgard 184, Dow Corning) was mixed with cross-linker in a 10 : 1 mass ratio and poured onto the prepared photoresist mold. The assembly was then degassed for 10 min, heated at 90°C for 2 h and demolded to form the soft template.

### 4.3. EDLs-Capacitive Pressure Sensor Preparation

The PDMS film with the slant hierarchical structure was imprinted using the C_4_F_8_-modified PDMS template after spin-coating and curing at 80°C. After demolding, the slant scales were treated by oxygen plasma and then pressed onto the bead template which was pre-filled with UV glue (NOA71, Norland Products Inc.) by blade coating. The assembly was exposed to UV light to obtain the hierarchical architecture, which was deposited with 20 nm Cr and 100 nm gold by magnetron sputtering to form the top electrode. The flat bottom electrode was obtained by depositing 20 nm Cr and 100 nm gold on a polyethylene terephthalate (PET) film with a thickness of 50 *μ*m. Subsequently, polyvinyl alcohol (PVA-210, Aladdin Industrial Corporation), [EMIM]OTF (98%, Aladdin Industrial Corporation), and deionized water at a mass ratio of 1 : 1.5 : 5.5 were stirred for 1 h in a water bath at 60°C and the mixed solution was then spin-coated on the bottom electrode and heated for 1 h to prepare the ion gel and bottom electrode assembly. This assembly was then overlapped with the PET-attached top electrode and sandwiched polyimide tapes as the spacers, and finally, the edge was encapsulated with 3 M scotch tape to obtain the sensor.

### 4.4. Pseudocapacitive Pressure Sensor Preparation

The slant hierarchical electrode coated with MXene was prepared by spraying 0.5 mL MXene solution (Ti_3_C_2_T*_x_*; 5 mg·mL^−1^; 11 Technology Co., Ltd.) onto the Au-coated electrode. The PVA/KOH solution was prepared by dissolving 1.5 g PVA in 10 mL deionized water at 60°C with magnetic stirring for 1 h, followed by adding 5 mL KOH solution with concentration of 0.3 g·mL^−1^ drop by drop slowly. The solution was then spin-coated on the flat MXene/Au-coated electrode to obtain the electrolyte and bottom electrode assembly. This assembly was then overlapped with the top electrode and sandwiched polyimide tapes as the spacers, and finally, the edge was encapsulated with 3 M scotch tape to obtain the sensor.

### 4.5. Magnetic Actuator Preparation

The PDMS film with porous structure was prepared by molding and the surface was modified by oxygen plasma treatment. The thickness of the film could be controlled by adjusting the spin coating speed. After ball milling for 20 h, the NdFeB (MQP-B, Magnequench, China) powders was mixed with ethanol (99.8%, Sigma-Aldrich) and coupling agent (KH560, Aladdin Industrial Corporation) in a 10 : 10 : 1 mass ratio and stirred until a uniform mixture was obtained, which was then filled into the holes by printing. This was followed by the drying, curing, and packaging phases, and finally, a magnetic film with discretized magnets was obtained. The film could be cut into the desired dimensions using a laser cutter.

### 4.6. Characterization

The structural morphology was examined by SEM (SU8010, HITACHI). Before imaging with SEM, a thin layer of gold (~10 nm) was coated on the samples. Capacitance measurements were performed at 1 kHz using a semiconductor analyzer (B1500A, Agilent). A highly configurable single-column testing machine (ESM303, Mark-10) was used to apply dynamic pressure. The frequency and magnitude of the load could be controlled by adjusting the speed and displacement. A force gauge (M5-10, Mark-10) was utilized to record the force. Frequency response tests were performed using a vibration exciter (SINOCERA JZK-10) to apply the periodic excitations. The sinusoidal signal generated by the signal generator (Agilent 33220A) is amplified by the power amplifier (SINOCERA YE5871A) and controls the vibration exciter to apply periodic pressure to the sensor. The piezoelectric signal generated by the impedance head (SINOCERA CL-YD-331) at the end of the exciter can be displayed by the oscilloscope (Tektronix DPO3034) after being amplified by the charge amplifier (ECON MI-2004-2). The applied pressure can be adjusted by adjusting the magnification of the power amplifier in conjunction with the oscilloscope display. In addition, a high-speed camera was used to capture the oscillation of the water droplets on the surface of the sensor and the deformation of the magnetic film at a frame rate of 4000 fps. A 30 V DC power supply was used to power the electromagnet. A precision balance was used to measure the magnetic force of the magnetic film.

### 4.7. Feedback and Control

Capacitance measurements and feedback were performed using custom-made electronics (Figure S22). The capacitance signal was acquired by means of a commercial capacitance-to-digital converter (CDC) (PCAP02AE, ACAM) with a measurement frequency of up to 500 kHz and an accuracy of up to 1 fF, whose test frequency was set to 1200 Hz in this experiment. A single-chip microcomputer (STM32, STMicroelectronics) was selected to control the CDC, assess the capacitance value, complete the setting of the output port depending on the capacitance signal, control the on and off function of the relay, and operate the electromagnet power supply circuit.

## Data Availability

All data needed to evaluate the conclusions in the paper are presented in the paper and/or the Supplementary Materials. Additional data related to this paper may be requested from the authors.

## Supplementary Materials

Supplementary Materials Supplementary 1: Figure S1: bioinspired slant hierarchical microstructure under different prepressures. Figure S2: controllable manufacturing of various hierarchical structures by the method of imprinting and transfer printing. Figure S3: ionic gel and the capacitance brought by EDLs. Figure S4: roughness of electrode surface and ionic gel surface characterized by AFM. Figure S5: in situ observation method for deformation process of slant structure. Figure S6: the deformation process of the slant flat scales. Figure S7: pseudocapacitive iontronic pressure sensor with the slant hierarchical electrode. Figure S8: sensing performance under different tangential forces. Figure S9: capacitance response to bending. Figure S10: comparison of the sensitivity corresponding to different pressure ranges. Figure S11: frequency response. Figure S12: consistency of sensors in different batches. Figure S13: slant electrode after five thousand loading/unloading cycles. Figure S14: response performance of sealed and unsealed sensors in high humidity environments. Figure S15: long-term performance of sealed and unsealed sensors at ambient temperature and humidity. Figure S16: capacitance response difference under different temperature. Figure S17: the minimum micropressure that can be detected. Figure S18: the super-hydrophobic surface of the sensor. Figure S19: manufacturing process of magnetic actuated trap. Figure S20: propertied of the magnetic actuated trap. Figure S21: Grabbing the quail egg without sensitive feedback. Figure S22: self-developed capacitor acquisition and feedback circuit. Supplementary 2: Supplementary Movie S1: dropping process of water onto the sensor surface. Supplementary 3: Supplementary Movie S2: biomimetic flytrap autonomously catching a dandelion. Supplementary 4: Supplementary Movie S3: biomimetic flytrap autonomously catching a bee. Supplementary 5: Supplementary Movie S4: sensitive flytrap grasping a quail egg without damage. Supplementary 6: Supplementary Movie S5: flytrap with dull sensitive feedback grasping a quail egg and damaging it.
