# Supplementary material for: Gecko-Inspired Slant Hierarchical Microstructure-Based Ultrasensitive Iontronic Pressure Sensor for Intelligent Interaction
Source: Research (Wash D C). 2022 Jun 14;2022:9852138. doi: 10.34133/2022/9852138 (PMC9275085; doi:10.34133/2022/9852138)
Supplement: Supplementary Materials — Supplementary 1: Figure S1: bioinspired slant hierarchical microstructure under different prepressures. Figure S2: controllable manufacturing of various hierarchical structures by the method of imprinting and transfer printing. Figure S3: ionic gel and the capacitance brought by EDLs. Figure S4: roughness of electrode surface and ionic gel surface characterized by AFM. Figure S5: in situ observation method for deformation process of slant structure. Figure S6: the deformation process of the slant flat scales. Figure S7: pseudocapacitive iontronic pressure sensor with the slant hierarchical electrode. Figure S8: sensing performance under different tangential forces. Figure S9: capacitance response to bending. Figure S10: comparison of the sensitivity corresponding to different pressure ranges. Figure S11: frequency response. Figure S12: consistency of sensors in different batches. Figure S13: slant electrode after five thousand loading/unloading cycles. Figure S14: response performance of sealed and unsealed sensors in high humidity environments. Figure S15: long-term performance of sealed and unsealed sensors at ambient temperature and humidity. Figure S16: capacitance response difference under different temperature. Figure S17: the minimum micropressure that can be detected. Figure S18: the super-hydrophobic surface of the sensor. Figure S19: manufacturing process of magnetic actuated trap. Figure S20: propertied of the magnetic actuated trap. Figure S21: Grabbing the quail egg without sensitive feedback. Figure S22: self-developed capacitor acquisition and feedback circuit. Supplementary 2: Supplementary Movie S1: dropping process of water onto the sensor surface. Supplementary 3: Supplementary Movie S2: biomimetic flytrap autonomously catching a dandelion. Supplementary 4: Supplementary Movie S3: biomimetic flytrap autonomously catching a bee. Supplementary 5: Supplementary Movie S4: sensitive flytrap grasping a quail egg without damage. Supplementary 6: Suppleme [file 9852138.f1.zip › Luo_Supplementary Material 1.docx]

Supplementary Material for

**Gecko-Inspired Slant Hierarchical Microstructure-based Ultra-Sensitive Iontronic Pressure Sensor for Intelligent Interaction**

Yongsong Luo, Xiaoliang Chen*, Hongmiao Tian, Xiangming Li, Yangtianyu Lu, Yang Liu, Jinyou Shao*

*Corresponding Email: xiaoliangchen@mail.xjtu.edu.cn; jyshao@mail.xjtu.edu.cn


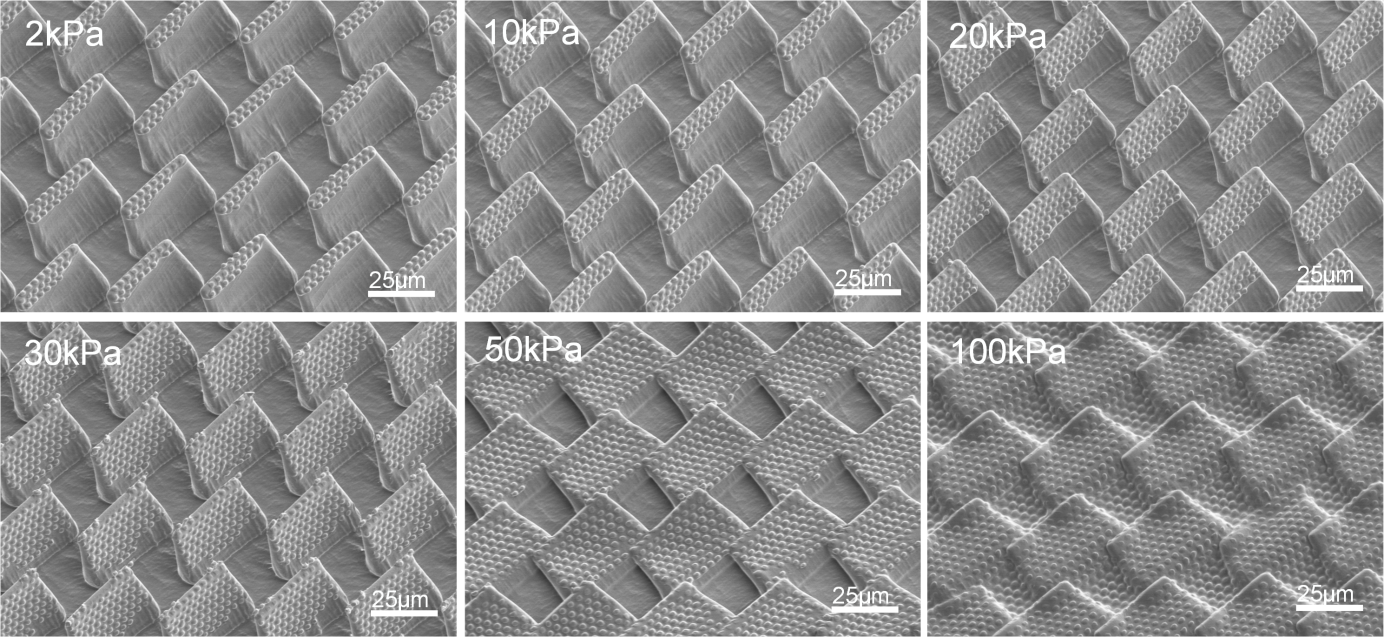


**Figure S1. Transfer printing results of the bioinspired slant hierarchical microstructure under different pre-pressures of 2 kPa, 10 kPa, 20 kPa, 30 kPa, 50 kPa and 100 kPa, respectively.**


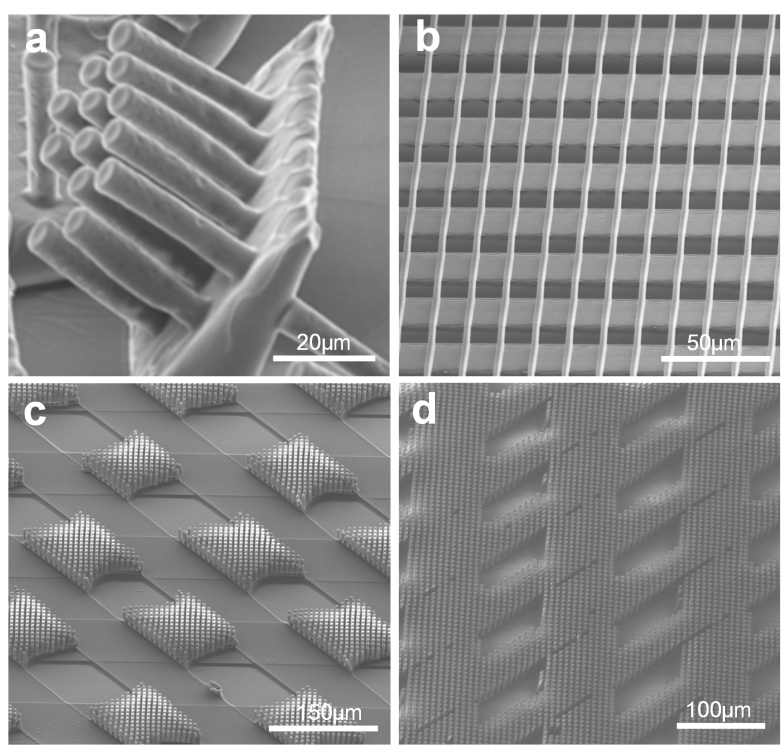


**Figure S2. Controllable manufacturing of various hierarchical structures by the method of imprinting and transfer printing. a** A large-aspect-ratio slant hierarchical structure closer the gecko’s feet. **b** Three-dimensional stacked grid lines. **c** and **d** Hierarchical structures with region selectivity on stacked grid lines.


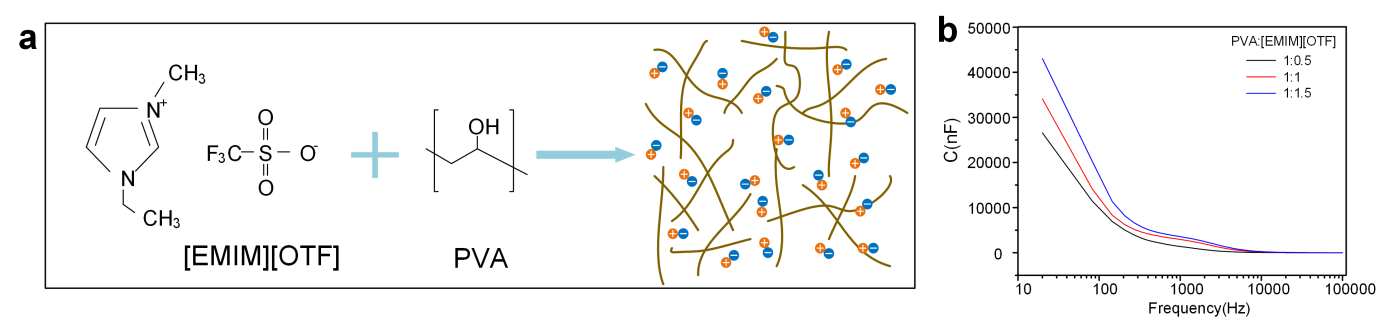


**Figure S3. Ionic gel and the capacitance brought by EDLs.** **a** The ionic gel composing ionic liquid and matrix: [EMIM]OTF and PVA. The gel was prepared by mixing ionic liquid, matrix and deionized water and then removing excess solvent. **b** Curves of the capacitance per unit area between the ion gel and the gold electrode versus frequency under different mass ratios. The higher the proportion of the ionic liquid, the larger the capacitance value. With the increase of the test frequency, limited by the ion migration rate, the measured value of capacitance decreased.


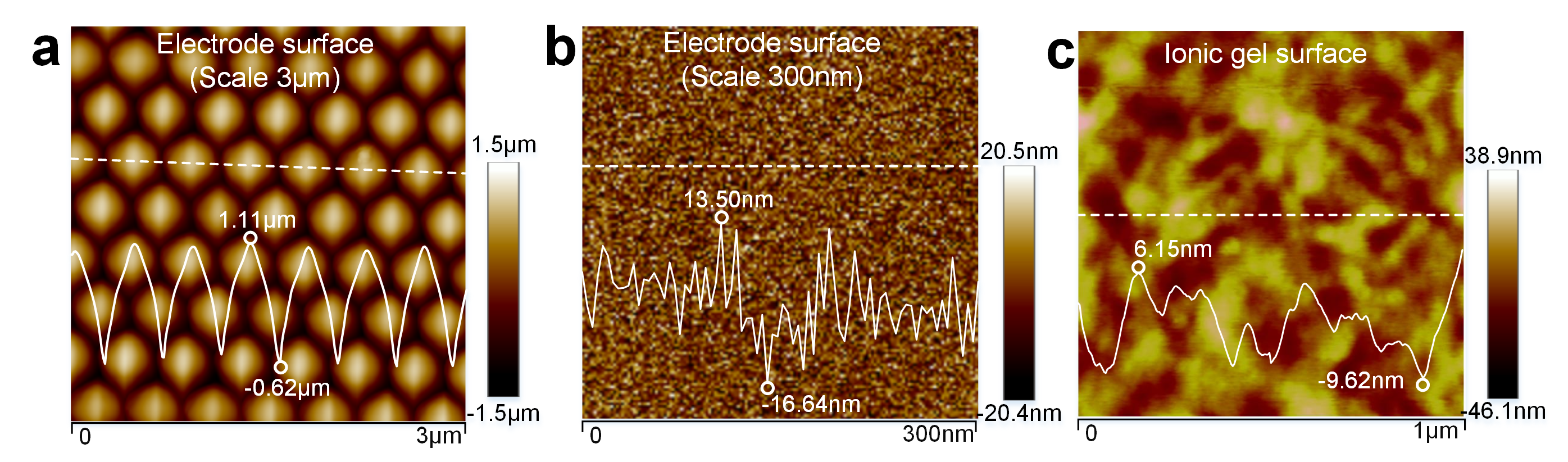


**Figure S4. Differences between simulation and experiment caused by the surface roughness.** Roughness of (**a**) and (**b**) electrode surface and (**c**) ionic gel surface characterized by AFM. The actual interfaces of ionic gel and Au-coated electrode previously treated with oxygen plasma were not as smooth as in the simulation but rather appear certainly rough. In the actual pressing process, the conformal fit at the ionic gel/electrode interface further brings much larger contact area than that shown in simulation.


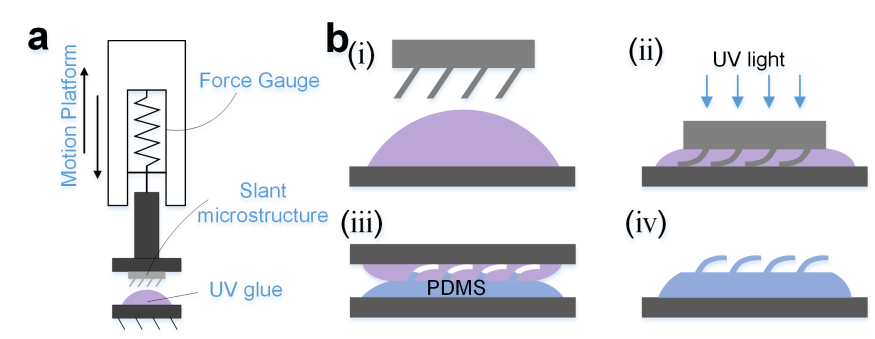


**Figure S5. In-situ observation method for deformation process of slant structure. a** Experiment equipment for preparing the sample for observation, including a motion platform and a force gauge. **b** Preparation process. (ⅰ) Apply a predetermined pressure on the slant structure and press it into the UV glue on a glass slide. (ⅱ) The top of the slant structure touches the glass slide and generates bending deformation, and the glue is cured by exposure to UV light, retaining the morphology of the bended structure. (ⅲ) After demolding, the PDMS is imprinted into the UV glue template. (ⅳ) After curing and demolding, the exactly same structure as the in-situ deformation is obtained.


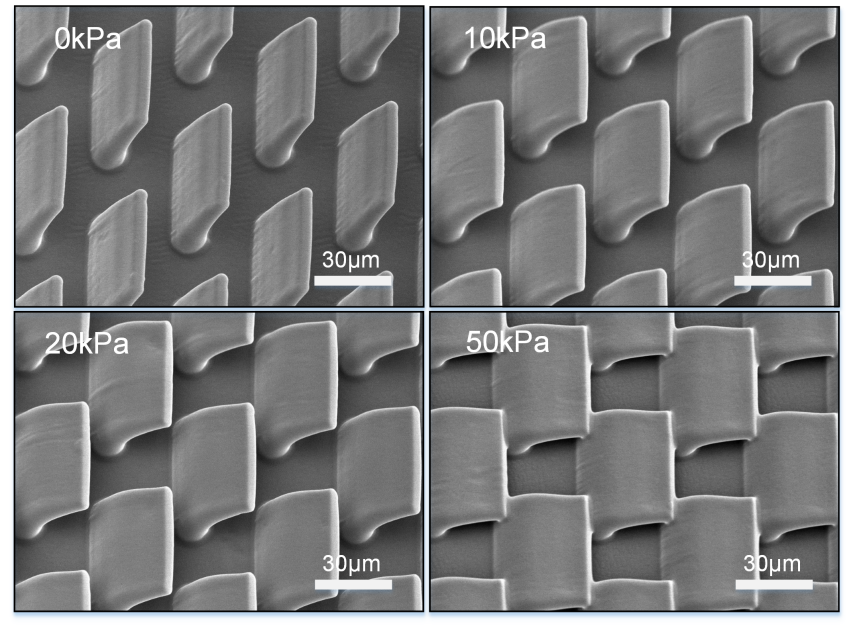


**Figure S6. The deformation process of the slant flat scales, similar to that of the slant hierarchical scales.**


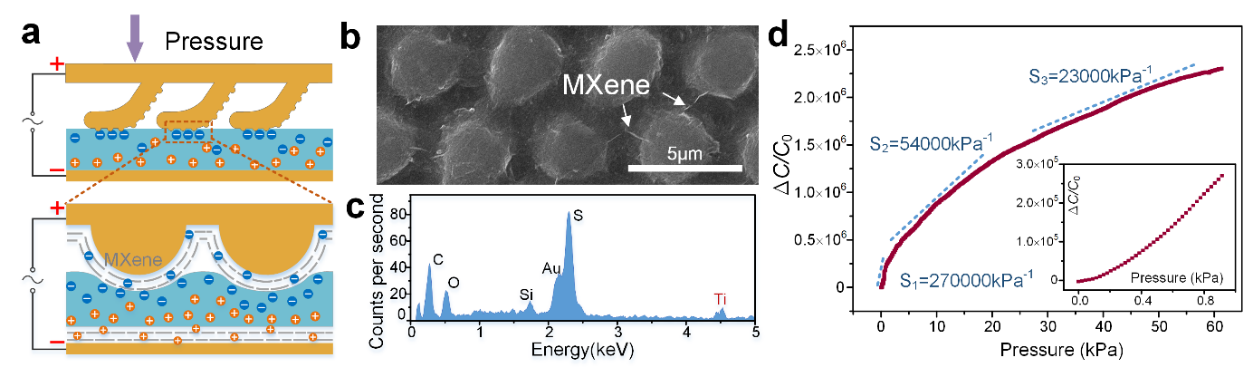


**Figure S7. Pseudocapacitive iontronic pressure sensor with the slant hierarchical electrode.** **a** Mechanism of the pseudocapacitive pressure sensor. The pseudocapacitance is primarily derived from the charges intercalated into the electrode, instead of just gathering on the electrode surface as in EDLs system, and thus the pseudocapacitance value is orders of magnitude higher than that of EDLs, thereby resulting in an even higher sensitivity. The slant hierarchical structure covered with low layer MXene (Ti3C2Tx) was used as the electrode and PVA/KOH gel was used as the electrolyte. When the slant scales are bent under pressure, the contact interface between the electrode and the electrolyte expands, and the charges not only gathers on the electrode surface, but also penetrates deeply into the interlayers of MXene. **b** SEM image of the MXene that coated on the slant hierarchical electrode. **c** Elemental analysis on the electrode. It can be seen that titanium was contained on the surface of the electrode, which only existed in MXene but not in the electrode material, demonstrating that the raised sheets on the surface of the electrode material were indeed MXene. **d** Sensing performance of the pseudocapacitive pressure sensor, with inset showing the range from 0.1 Pa to 1 kPa. A superior sensitivity of 270,000 kPa-1 (0-1 kPa), 54000 kPa-1 (1-20 kPa) and 23000 kPa-1 (>20 kPa) can be obtained, which is much higher than that in the previous report. Such a big promotion in sensitivity of pseudocapacitive sensor using the same strategy can be attributed to the large area changes produced by the slant hierarchical architecture, and this result also demonstrates the importance and the universality of our strategy for different capacitor systems.


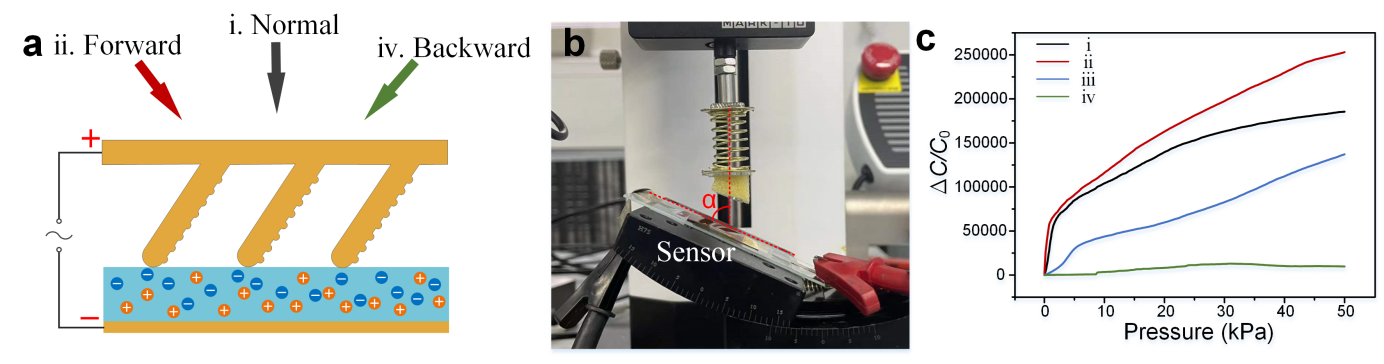


**Figure S8. Sensing performance under different tangential forces.** **a** The directions of the tested forces. Four different forces were applied to the sensor, respectively, including normal force, forward force, backward force and orthogonal force. The ⅱ-forward force and ⅳ-backward force were along the slant direction and the ⅲ-force, which is not shown in the figure, was orthogonal to the slant direction, that is, in the plane perpendicular to the schematic diagram. **b** Experiment construction. An indenter with an inclined plane was prepared by cutting the sponge. The sensor was fixed on the goniometer and then the goniometer was adjusted parallel to the surface of the indenter. The inclined angle *α* was about 65°. The spring above the sponge can ensure that the indenter and the sensor surface can be fully fitted during the loading process. **c** Capacitance responses under forces with different directions. The forward force can push the slant scale to be flatten faster, so it has a higher sensitivity (the red curve) at the beginning compared to that under the normal force (the black curve), but the pressure regime with highest sensitivity is reduced. Due to a certain amount of friction, the backward force may cause the slant scale to bend to the other side. The process is not stable and will not effectively enhance the contact interface area. The effect of ⅲ-force is similar to the normal force, but because the normal component only occupies a part, its capacitance curve is lower than that of the normal force, as shown in the blue curve.


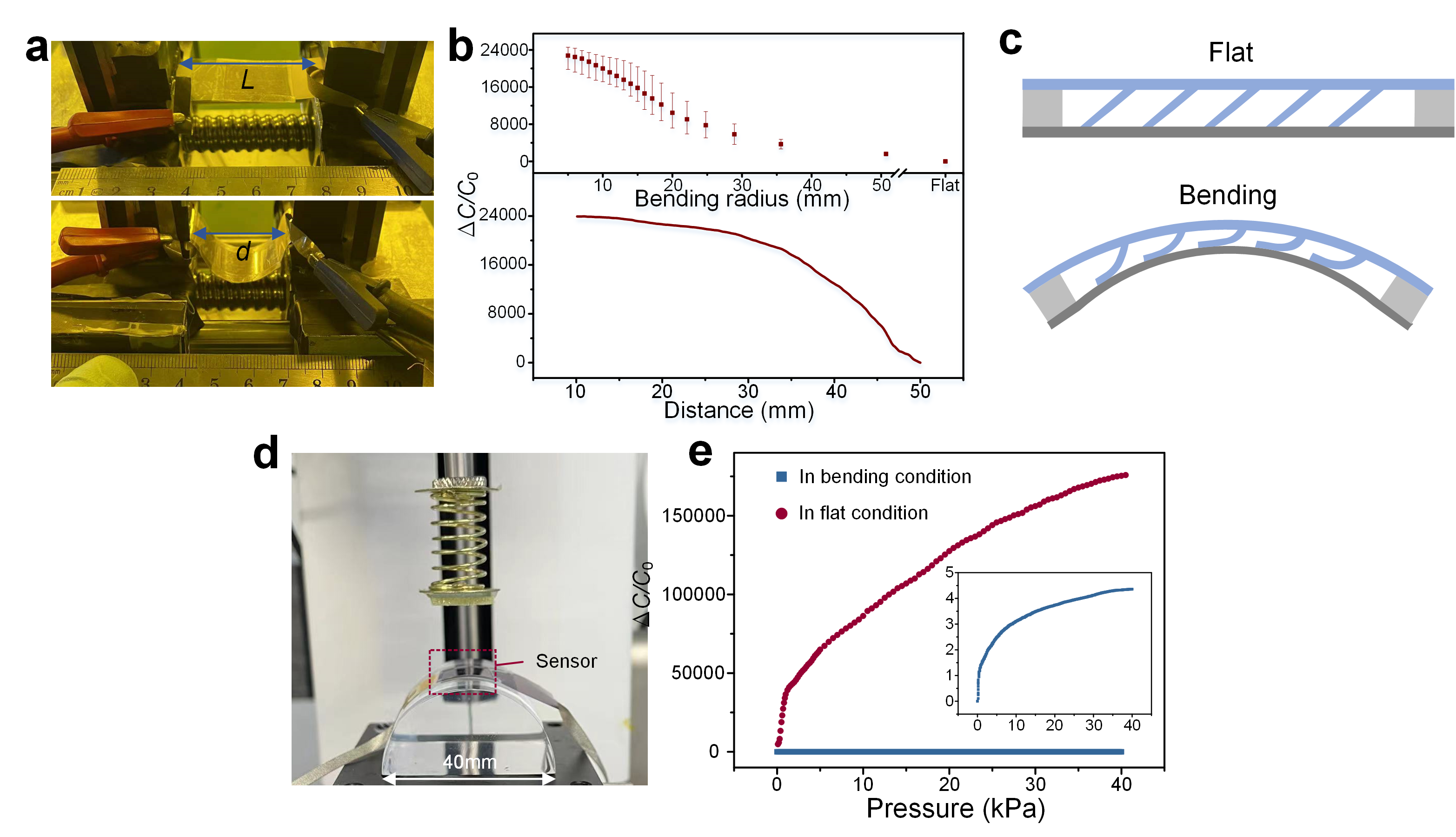


**Figure S9. Capacitance response to bending. a** Bending test configuration. The sensor was fixed on the stretching platform, and the initial length was 50 mm. One clamp of the platform was fixed, and the other one carried one end of the sensor to move horizontally. When the movable end approached the other one, the sensor was bent. **b** The capacitance response in the process of uniformly reducing the distance between two clamps to 10 mm. **c** The squeezing of the upper and lower electrodes during bending leading to pre-deformation of the slant scale, thus resulting in increased capacitance. **d** Diagram of the pressure loading device in the bending state. The sensor was attached to a cylindrical surface with a radius of 20 mm, and pressure was applied to the sensor through the platform. **e** Capacitive response of the sensor in the bending condition, as shown in inset, compared with that in the flat condition. Since the initial capacitance value of the sensor has been increased by about 10,000 times after bending, the relative capacitance change caused by external pressures exhibits an order of magnitude decrease.


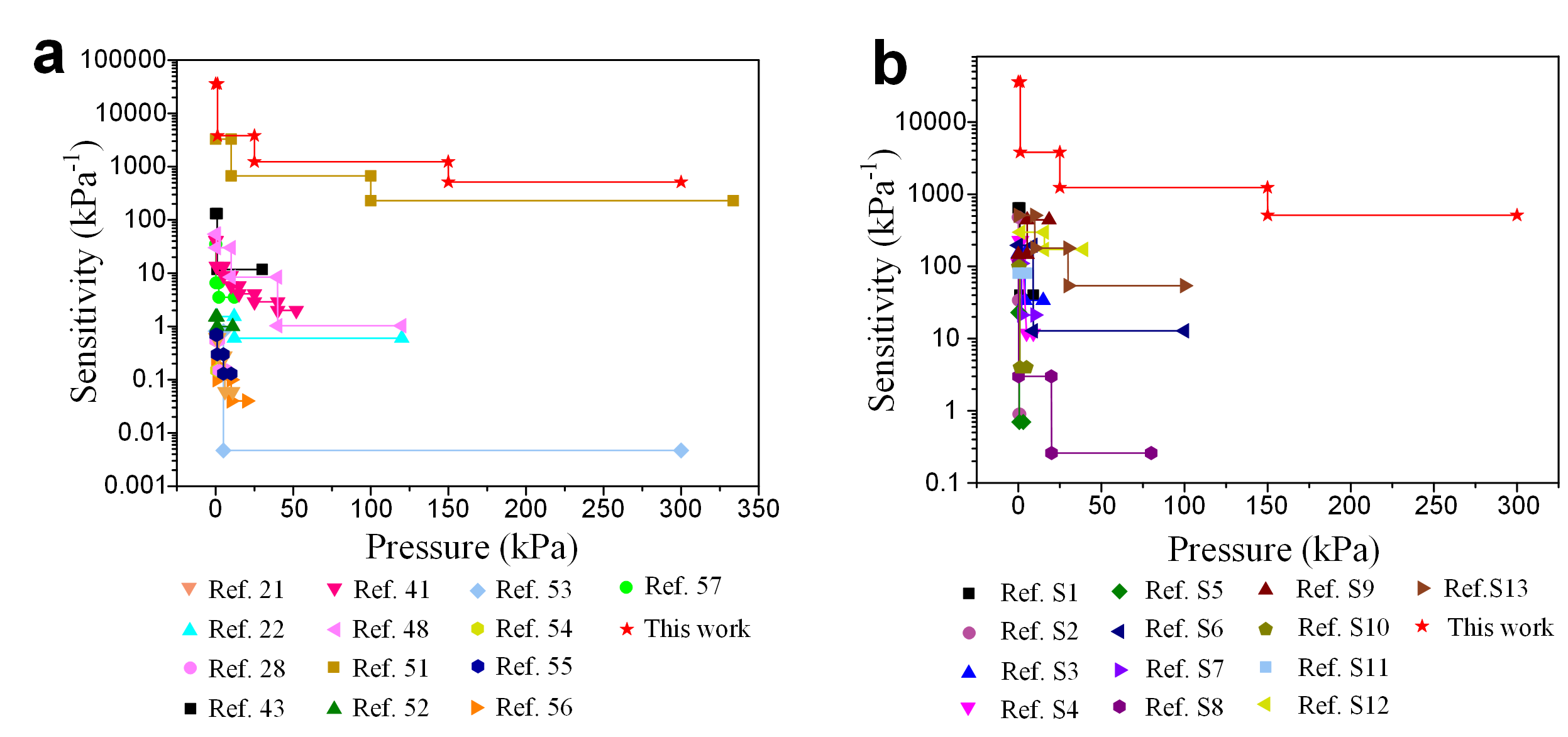


**Figure S10. Comparison of the sensitivity corresponding to different pressure ranges. a** Comparison between the proposed sensor and capacitive pressure sensors reported in the literature. **b** Comparison between the proposed sensor and piezoresistive sensors reported in the literature [1-13].


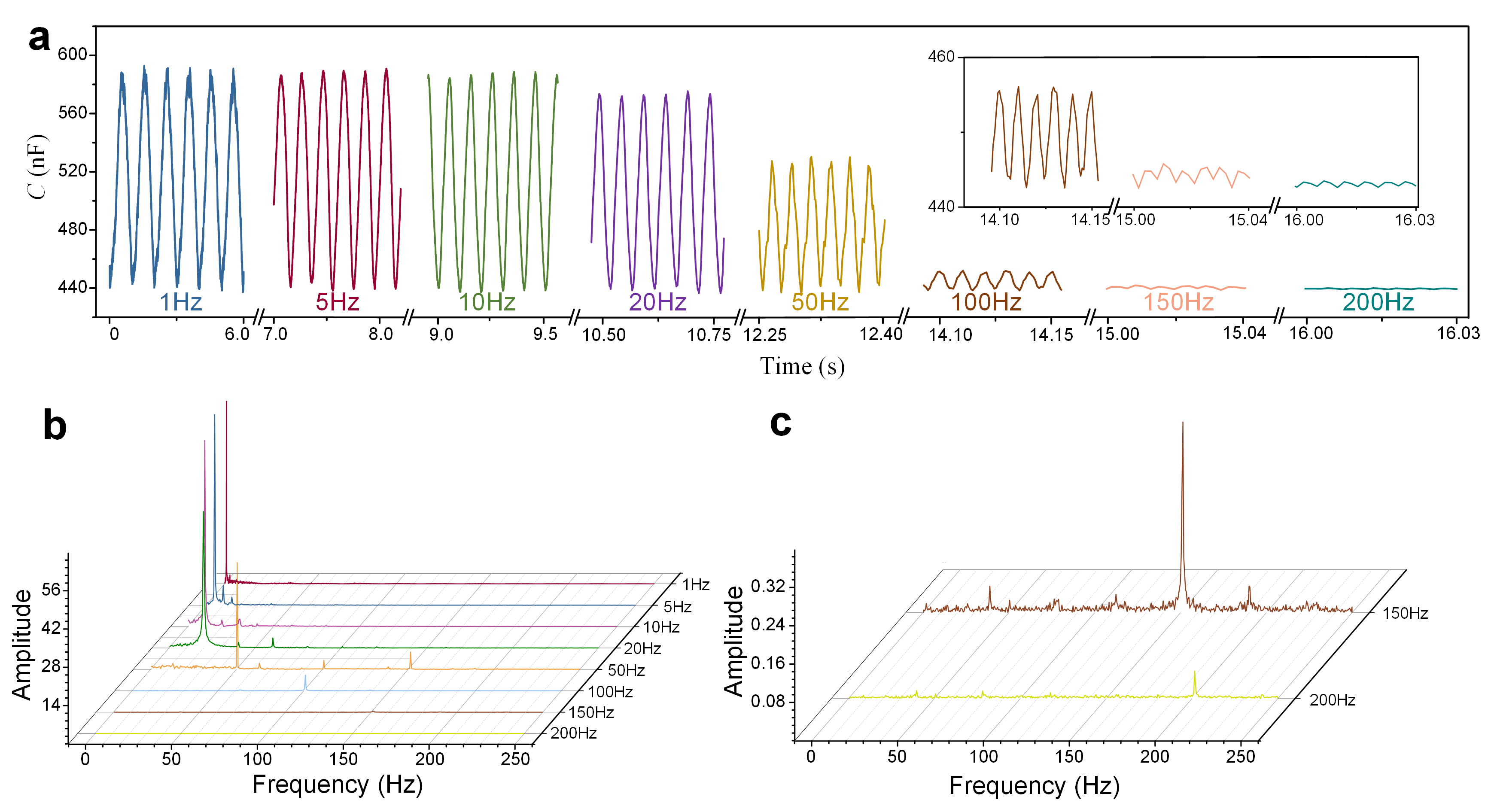


**Figure S11. Frequency response. a** Time-domain signals of capacitive response to periodic pressures of about 0.4 N at frequencies of 1 Hz, 5 Hz, 10 Hz, 20 Hz, 50 Hz, 100 Hz, 150 Hz and 200 Hz, with fixing the sensor on the lift table of the exciter and preloading a force of about 0.2 N to ensure that the sensor and the head were in contact during the whole excitation process. **b** and **c** Amplitude-frequency diagrams obtained by fast Fourier transform of the time-domain signals.


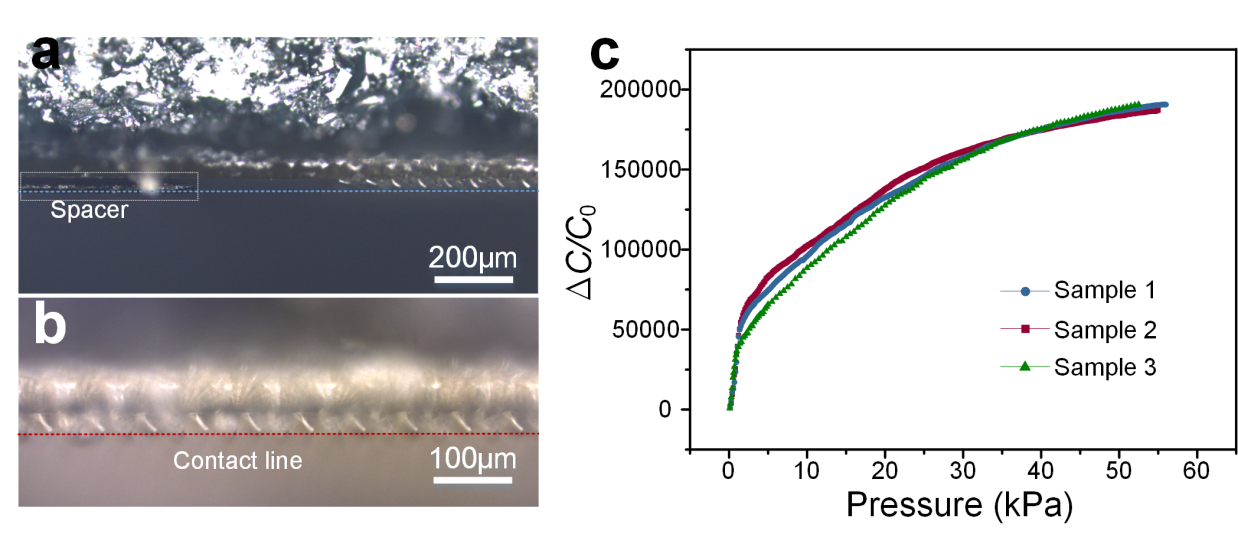


**Figure S12. Consistency of sensors in different batches. a** Photo of the sensor before package. **b** Photo of the contact line between the electrode and gel after package. Due to the advantage of this method that it can ensure the accuracy and consistency of the microstructure, the sensors in different batches have highly consistent structures with the same height. Therefore, polyimide tape with the same thickness can be used as spacers to ensure that the sensors have the same initial capacitance. **c** Capacitance responses of three sensors in different batches.


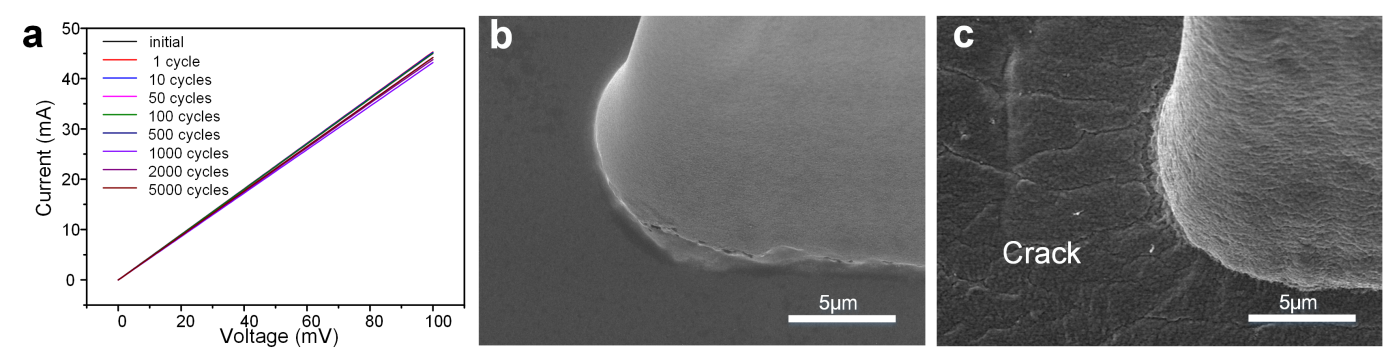


**Figure S13. Slant electrode after five thousand loading/unloading cycles.** **a** Conductivity of the structured electrode after bending. After 5000 cycles, the conductivity of the electrode decreased by less than 5%. **b** SEM image of the electrode before bending. **c** SEM image of the electrode after 5000 cycles. In the repeated process of deformation and recovery, the gold on the surface of the structure does produce certain cracks. However, due to the constrain of the periodic slant scales, the cracks will not cut through the whole electrode. When they extend to the boundary of the slant scale, the cracks propagation will be restrained. And because Cr or Ti was deposited as the bonding layer before depositing gold, although gold will crack, it will not peel off from the surface.


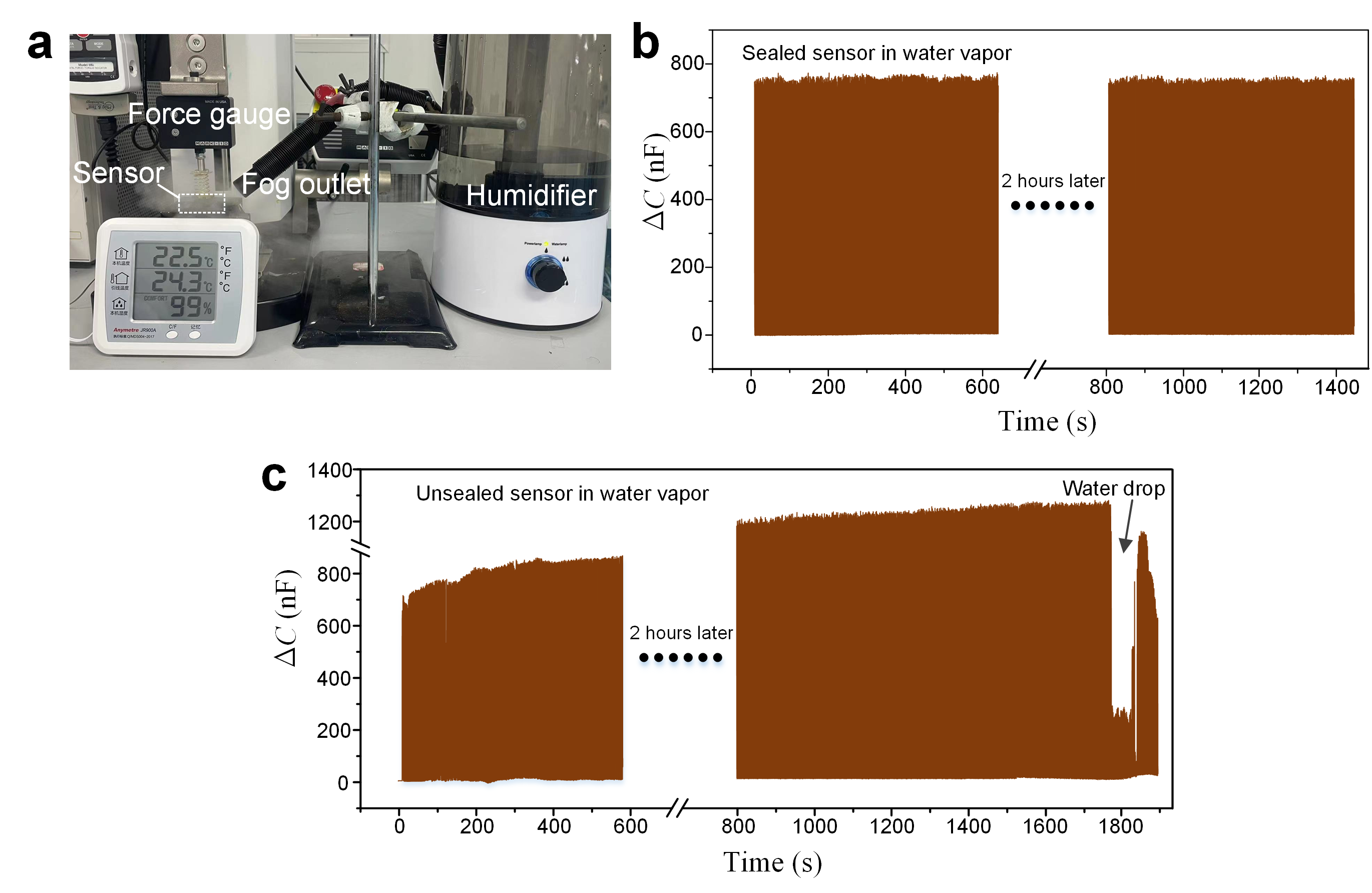


**Figure S14. Response performance of sealed and unsealed sensors in high humidity environments.** **a** The schematic diagram of the testing setup. The humidifier was used to continuously generate water vapor rushing towards the sensor to provide a high-humidity atmosphere. The sensors were loaded with a periodic pressure of about 10 kPa using the testing platform, and a hygrometer was placed next to the sensor to reflect the relative humidity in real time. **b** Capacitance change of the sealed sensor under a periodic pressure of 10 kPa, showing high consistency. **c** Capacitance change of the unsealed sensor under a periodic pressure of 10 kPa, showing a continuously increasing capacitance. After adding a water drop on the edge, the ionic gel layer dissolved and the capacitance changed sharply.


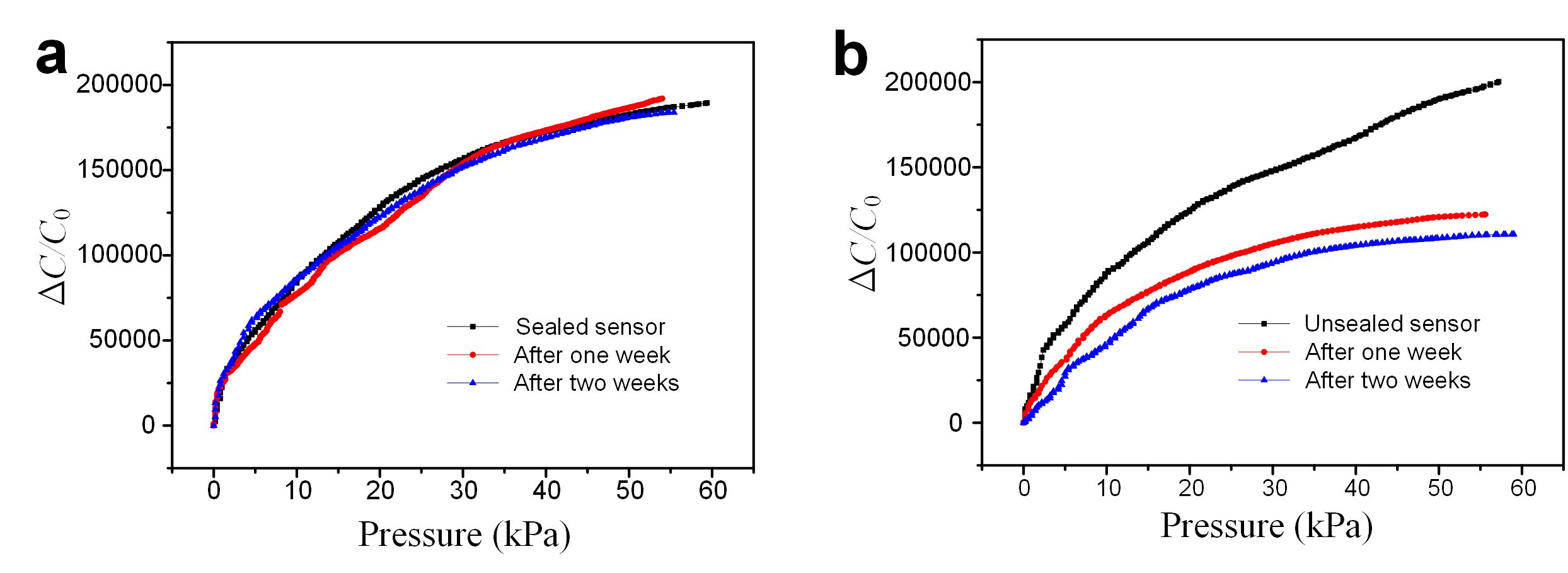


**Figure S15. Long-term performance of sealed and unsealed sensors at ambient temperature and humidity.** **a** The sealing method isolating the ionic gel from the environment, ensuring consistent sensor performance. **b** The ionic gel continuously losing moisture and tending to dry-up, resulting in attenuation of the capacitive response.


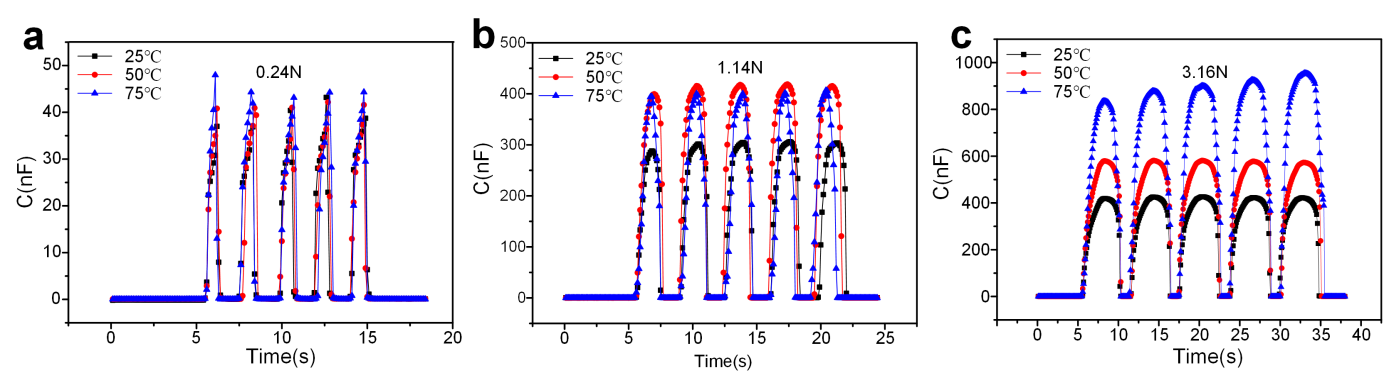


**Figure S16. Capacitance response difference under different temperature.** The increase in temperature leads to the increase in ion migration rate and therefore results in the increase in capacitance. At room temperature, the capacitance response has high stability. Since the sensitivity in the low pressure regime is much higher, the capacitance increases faster when the pressure is small. Therefore, the curve of 0.24 N has a sharper shape. When the pressure increases to 1.14 N and 3.16 N, the capacitance growth slows down, and thus the response curves become gentle.


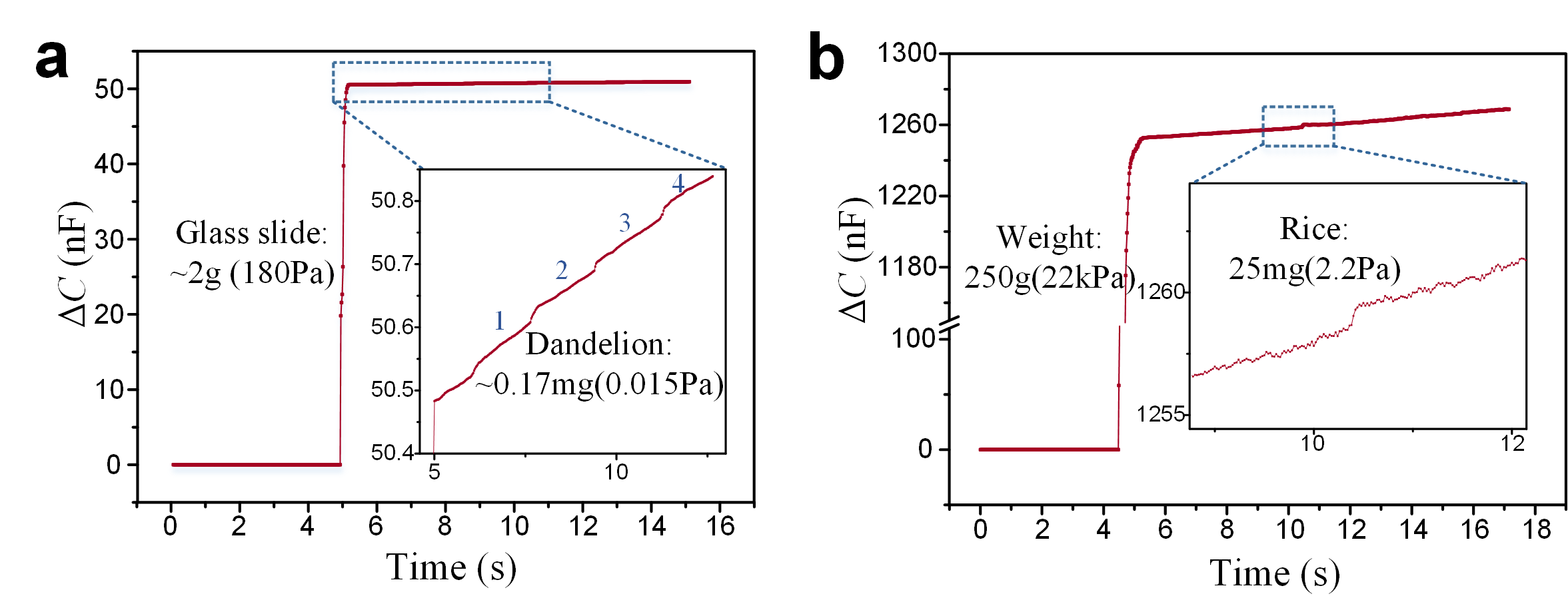


**Figure S17.** **The minimum micro pressure that can be detected.** **a** Sensor response to four dandelion seeds when preloaded with a small pressure. In the low pressure range (<1 kPa), the sensitivity of the sensor is ultra-high, so it has a clearer response to extremely small micro pressures which can reach to the LOD. **b** Sensor response to a grain of rice when preloaded with a high pressure. In the high pressure range, the sensitivity of the sensor decreases, and the minimum micro pressure that can be detected is far from reaching the LOD.


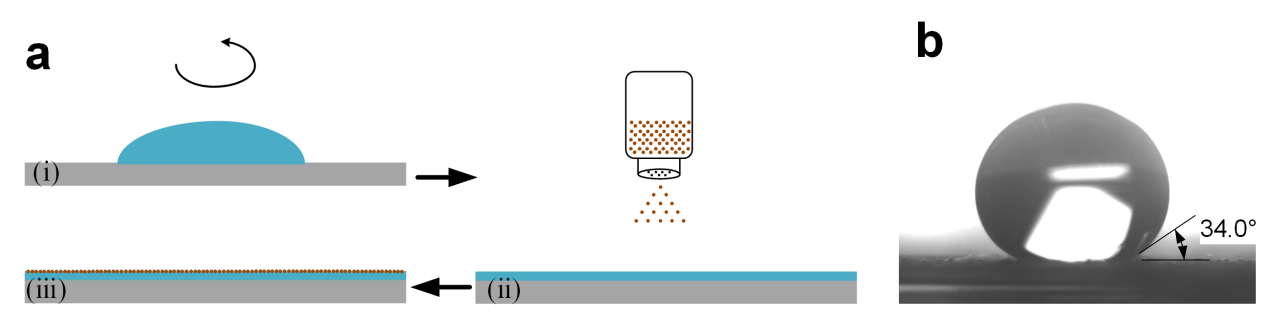


**Figure S18. The super-hydrophobic surface of the sensor.** **a** Manufacturing process of the super-hydrophobic surface. The sensor surface was spin-coated with PDMS as adhesive and then was sprinkled an even layer of nano powders to prepare the hydrophobic surface. **b** Water contact angle of 146° at this proposed surface.


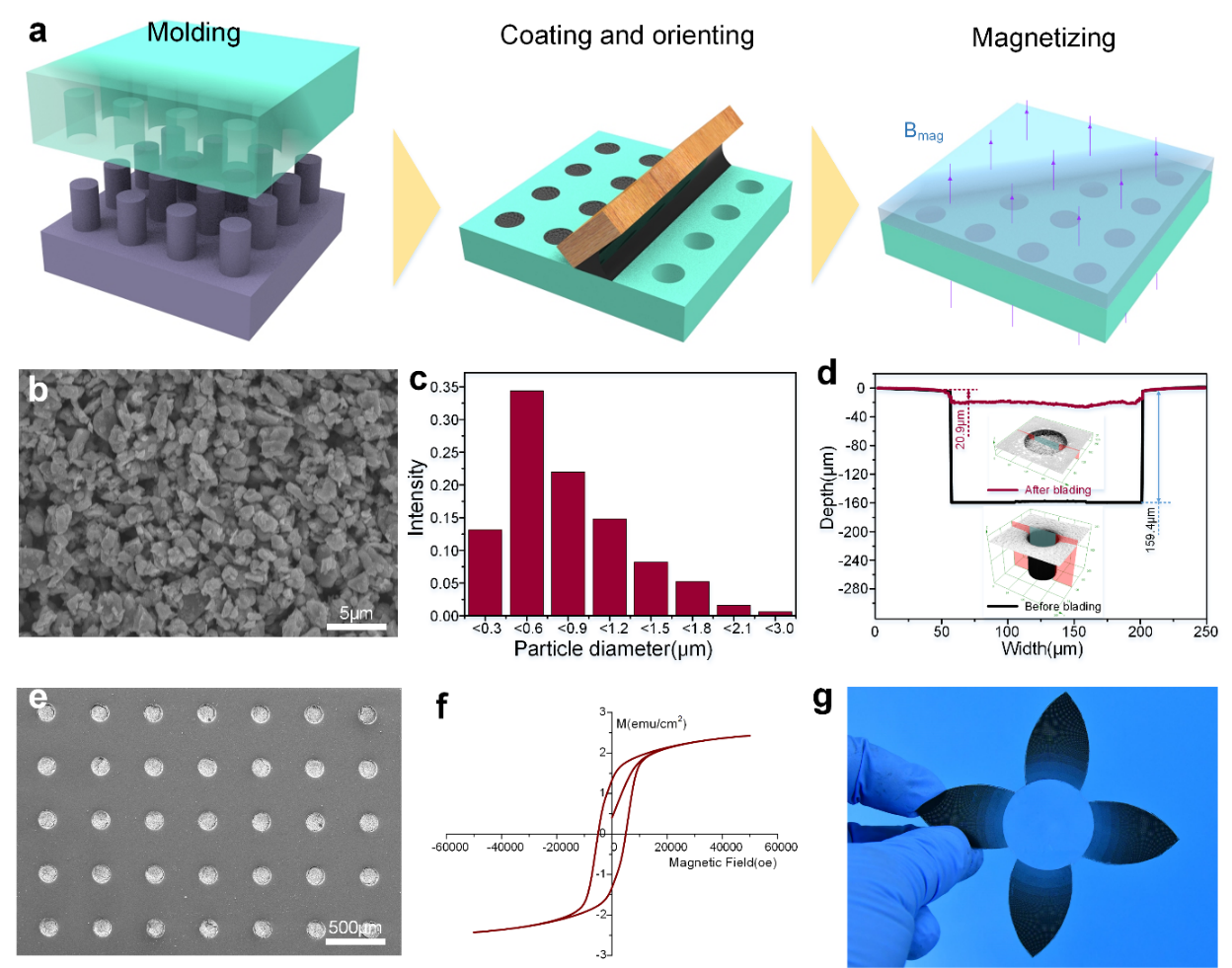


**Figure S19. Manufacturing process of magnetic actuated trap.** **a** Magnetic film preparation process, including mold imprinting, embedded printing of magnetic particles, encapsulation, and magnetization. The PDMS film with porous structure was prepared by molding and the surface was modified by oxygen plasma treatment. The NdFeB powders after ball milling for 20h was mixed with ethanol and coupling agent in a 10:10:1 mass ratio to prepare the ink. And then the ink was filled into the holes by printing. This was followed by the drying, curing, and packaging phases, and finally, a magnetic film with discretized magnets was obtained. **b** SEM image of the milled NdFeB powder. The mass ratio of corundum balls, NdFeB particles and ethanol is 2:1:1. **c** The particle size distribution of NdFeB powder. **d** Remaining depth of the polymer template holes before and after the printing of magnetic powders. **e** SEM image of the filled holes. **f** Hysteresis loop of the printed film in a magnetic field with a maximum intensity of 5T. **g** Magnetically-actuated trap.


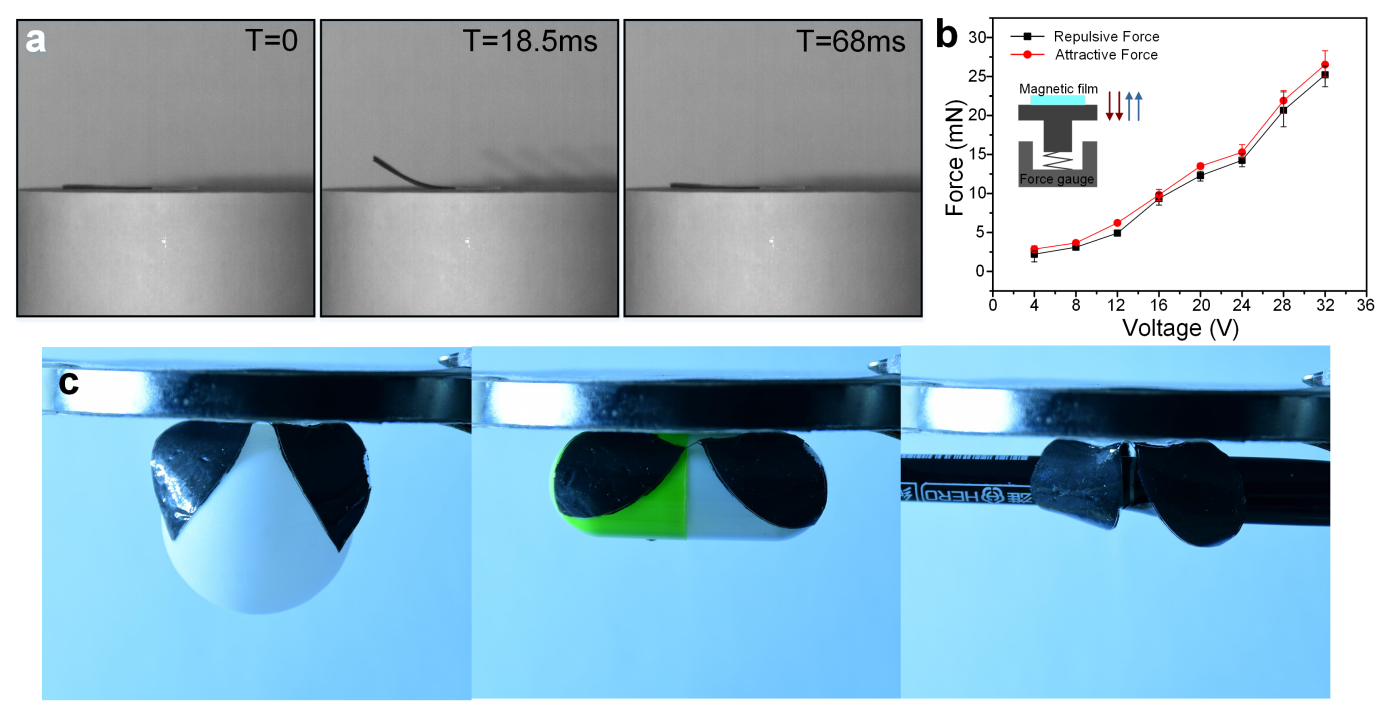


**Figure S20. Propertied of the magnetic actuated trap. a** Deformation process of magnetic films in the magnetic field with the strength of 35 Mt, with the response time of 18.5 ms and recover time of 50ms, which are faster than those of Venus flytrap. **b** The magnetic force generated by the actuated trap under different driving voltages. **c** The magnetic trap is able to grab a ping-pang, a capsule, and a pen.


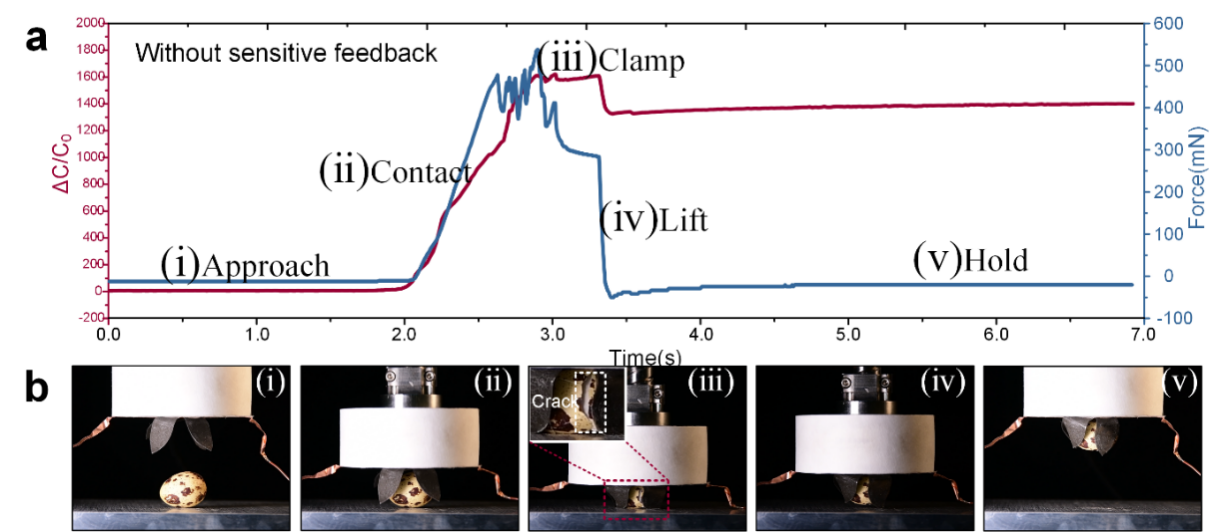


**Figure S21. Grabbing the quail egg.** **a** The capacitance and force change during the process of grabbing a quail egg using the flytrap without sensitive perception. **b** Photos show the grabbing process.


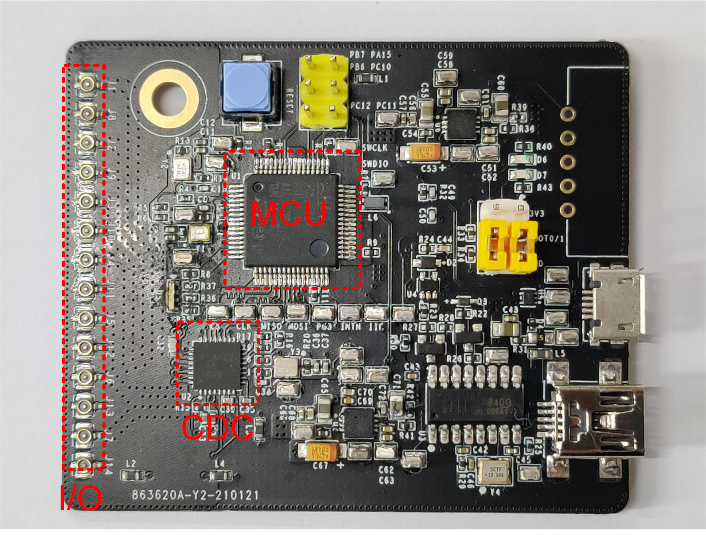


**Figure S22. Self-developed capacitor acquisition and feedback circuit.**

[1] L. Bi, Z. Yang, L. Chen, Z. Wu and C. Ye. Compressible AgNWs/Ti3C2Tx MXene aerogel-based highly sensitive piezoresistive pressure sensor as versatile electronic skins. Journal of Materials Chemistry A 8 (2020) 20030-20036.

[2] Y. Zhu, H. Cai, H. Ding, N. Pan and X. Wang. Fabrication of Low-Cost and Highly Sensitive Graphene-Based Pressure Sensors by Direct Laser Scribing Polydimethylsiloxane. ACS Appl Mater Interfaces 11 (2019) 6195-6200.

[3] Y. Cheng, Y. Ma, L. Li, M. Zhu, Y. Yue, W. Liu, L. Wang, S. Jia, C. Li, T. Qi, et al. Bioinspired Microspines for a High-Performance Spray Ti3C2Tx MXene-Based Piezoresistive Sensor. ACS Nano 14 (2020) 2145-2155.

[4] C. Luo, N. Liu, H. Zhang, W. Liu, Y. Yue, S. Wang, J. Rao, C. Yang, J. Su, X. Jiang, et al. A new approach for ultrahigh-performance piezoresistive sensor based on wrinkled PPy film with electrospun PVA nanowires as spacer. Nano Energy 41 (2017) 527-534.

[5] B. Zhu, Y. Ling, L.W. Yap, M. Yang, F. Lin, S. Gong, Y. Wang, T. An, Y. Zhao and W. Cheng. Hierarchically Structured Vertical Gold Nanowire Array-Based Wearable Pressure Sensors for Wireless Health Monitoring. ACS Appl Mater Interfaces 11 (2019) 29014-29021.

[6] Z. Wang, L. Zhang, J. Liu, H. Jiang and C. Li. Flexible hemispheric microarrays of highly pressure-sensitive sensors based on breath figure method. Nanoscale 10 (2018) 10691-10698.

[7] W. Liu, N. Liu, Y. Yue, J. Rao, C. Luo, H. Zhang, C. Yang, J. Su, Z. Liu and Y. Gao. A flexible and highly sensitive pressure sensor based on elastic carbon foam. Journal of Materials Chemistry C 6 (2018) 1451-1458.

[8] K. Xia, C. Wang, M. Jian, Q. Wang and Y. Zhang. CVD growth of fingerprint-like patterned 3D graphene film for an ultrasensitive pressure sensor. Nano Research 11 (2017) 1124-1134.

[9] Y. Yue, N. Liu, W. Liu, M. Li, Y. Ma, C. Luo, S. Wang, J. Rao, X. Hu, J. Su, et al. 3D hybrid porous Mxene-sponge network and its application in piezoresistive sensor. Nano Energy 50 (2018) 79-87.

[10] Y. Gao, C. Yan, H. Huang, T. Yang, G. Tian, D. Xiong, N. Chen, X. Chu, S. Zhong, W. Deng, et al. Microchannel‐Confined MXene Based Flexible Piezoresistive Multifunctional Micro‐Force Sensor. Advanced Functional Materials 30 (2020).

[11] Y. Hu, H. Zhuo, Q. Luo, Y. Wu, R. Wen, Z. Chen, L. Liu, L. Zhong, X. Peng and R. Sun. Biomass polymer-assisted fabrication of aerogels from MXenes with ultrahigh compression elasticity and pressure sensitivity. Journal of Materials Chemistry A 7 (2019) 10273-10281.

[12] M. Chao, L. He, M. Gong, N. Li, X. Li, L. Peng, F. Shi, L. Zhang and P. Wan. Breathable Ti3C2Tx MXene/Protein Nanocomposites for Ultrasensitive Medical Pressure Sensor with Degradability in Solvents. ACS Nano 15 (2021) 9746-9758.

[13] L. Yang, H. Wang, W. Yuan, Y. Li, P. Gao, N. Tiwari, X. Chen, Z. Wang, G. Niu and H. Cheng. Wearable Pressure Sensors Based on MXene/Tissue Papers for Wireless Human Health Monitoring. ACS Appl Mater Interfaces 13 (2021) 60531-60543.
